# Supplementary material for: Efficacy of Kan Jang® in Patients with Mild COVID-19: Interim Analysis of a Randomized, Quadruple-Blind, Placebo-Controlled Trial
Source: Pharmaceuticals (Basel). 2022 Aug 17;15(8):1013. doi: 10.3390/ph15081013 (PMC9415141; doi:10.3390/ph15081013)
Supplement: Supplementary file 1 [file pharmaceuticals-15-01013-s001.zip › Supplement S2.pdf]

Duration of mild COVID-19 symptoms over the time of treatment and follow up

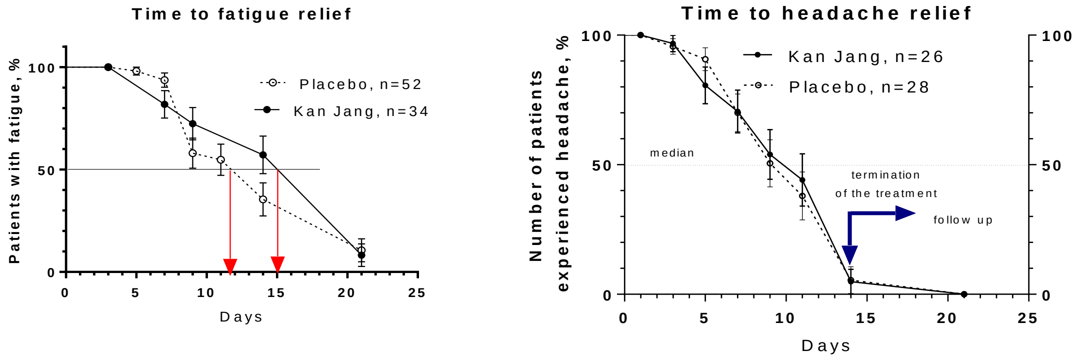

Figure S1. Kaplan-Meier curves show the number of participants who experienced the symptoms of mild COVID over the time from randomization (Day 1) to the end of the treatment (Day 14) and followed up for one week (Day 21): (a) - fatigue, (b) – pain.

Relief of severity of the mild COVID-19 symptoms the time of treatment and follow up

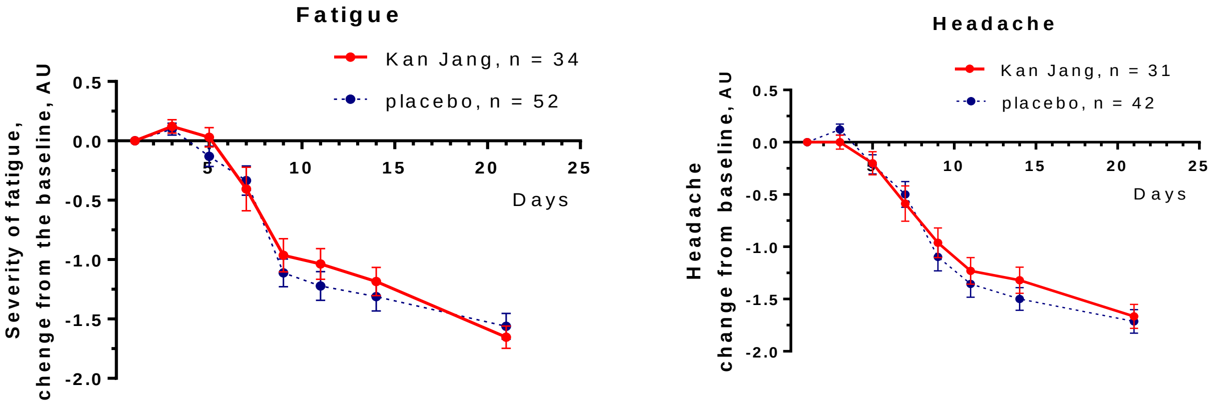

Figure S2. (a) - Fatigue scores (mean ± SEM) of patients in group A (Kan Jang) and group B (placebo) over the time from Day 1 to Day 21. The changes from the baseline within groups A and B over time were significant ($p<0.0001$); between-groups comparison of the changes of fatigue score from the baseline over time shows no interaction (p=0.8002), and no significant difference (p =0.2460) between groups A and B. The Kan Jang treatment has no statistically significant effect on fatigue compared to placebo; (b) - Headache scores (mean ± SEM) of patients in group A (Kan Jang) and group B (placebo) over the time from Day 1 to Day 21. The changes from the baseline within groups A and B over time were significant ($p<0.0001$); between-groups comparison of the changes of headache score from the baseline over time shows no interaction (p=0.8303), and no significant difference (p =0.4993) between groups A and B. The Kan Jang treatment has no statistically significant effect on headache compared to placebo.

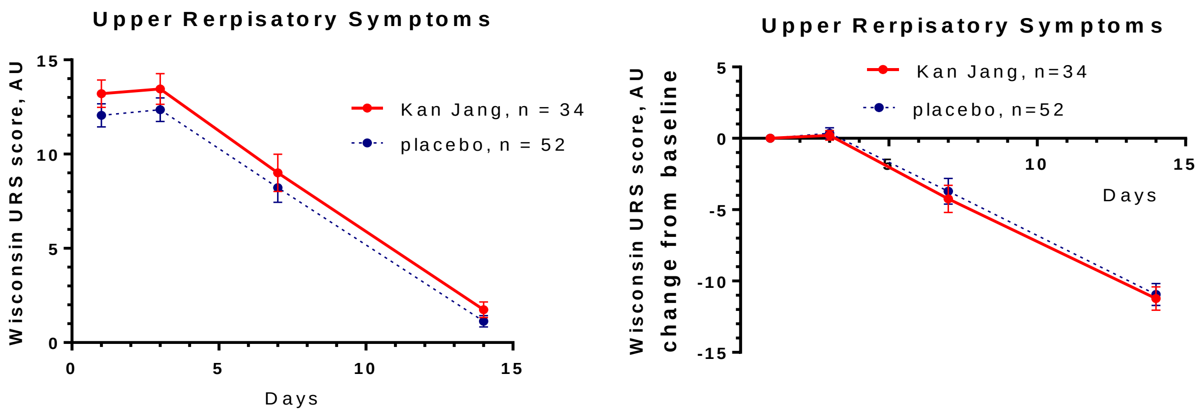

Figure S3. (a) – Wisconsin Upper Respiratory Symptom Survey Questionary scores (mean ± SEM) of patients in group A (Kan Jang) and group B (placebo) over the time from Day 1 to Day 14. The changes from the baseline within groups A and B over time were significant ($p<0.0001$; calculated by Repeated measures ANOVA); Two-way ANOVA calculated the significance of the interaction between treatment groups over time; $p= 0.9807$. (b) Between-groups comparison of the changes of HAMA score from the baseline over time shows no interaction (p= 0.9733), and no significant difference ($p = 0.5784$) between groups A and B. The Kan Jang treatment has no statistically significant effect on the Wisconsin Upper Respiratory Symptom Survey Questionary Score scores of patients compared to placebo.

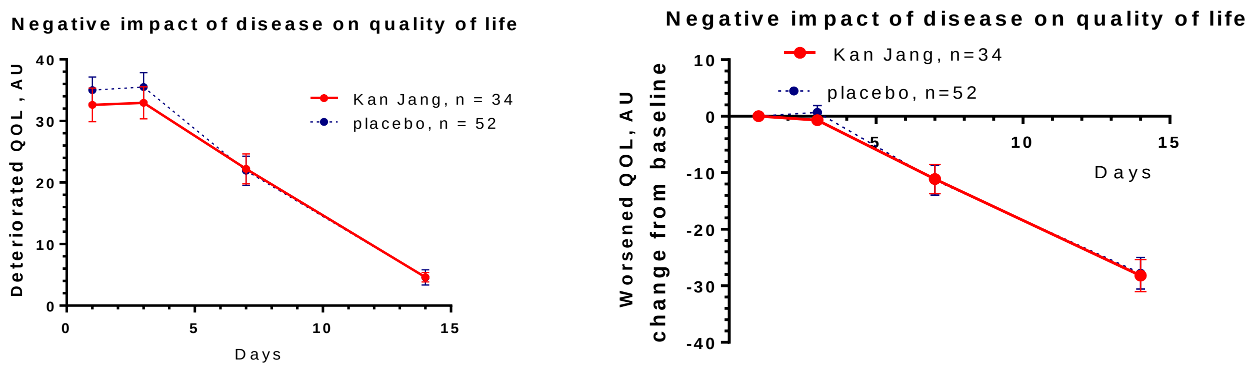

Figure S4. (a) – Wisconsin Upper Respiratory Symptom Survey Questionary Quality of Life scores (mean ± SEM) of patients in group A (Kan Jang) and group B (placebo) over the time from Day 1 to Day 14. The changes from the baseline within groups A and B over time were significant (p<0.0001; calculated by Repeated measures ANOVA); Two-way ANOVA calculated the significance of the interaction between treatment groups over time; p= 0.8862. (b) Between-groups comparison of the changes of HAMA score from the baseline over time shows no interaction (p= 0.9731), and no significant difference (p = 0.7705) between groups A and B. The Kan Jang treatment has no statistically significant effect on the Wisconsin Upper Respiratory Symptom Survey Questionary Quality of Life scores of patients compared to placebo.

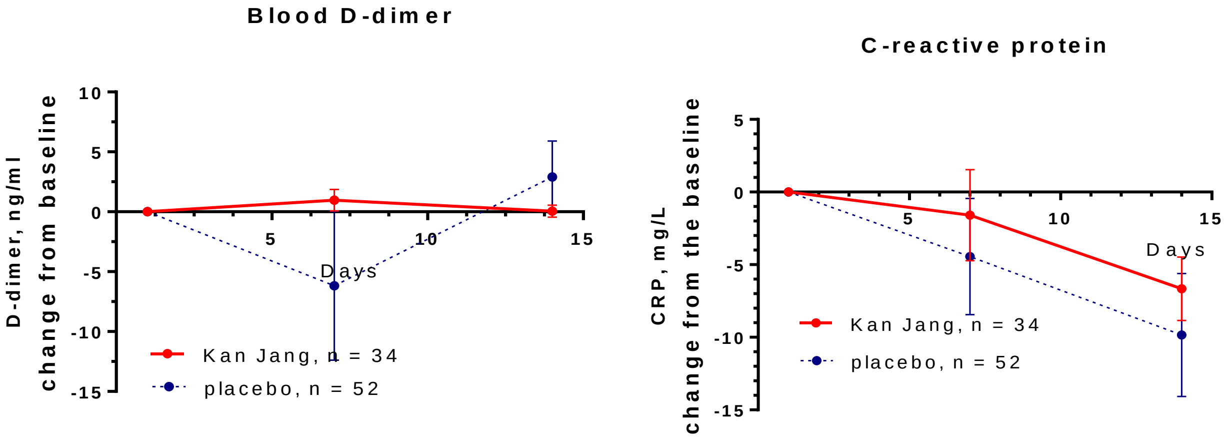

Figure S5. (a) – Blood serum D -dimer concentration (mg/L, mean ± SEM) of patients in group A (Kan Jang) and group B (placebo) over the time from Day 1 to Day 14. The changes from the baseline within groups A and B over time were insignificant (p>0.05); between-groups comparison of the changes of Blood serum D -dimer from the baseline over time shows no interaction (*p*=0.3195), and no significant difference (*p* =0.6051) between groups A and B. The Kan Jang treatment has no statistically significant effect on blood serum D -dimer concentration compared to placebo**.** (b) – Blood serum C-reactive protein concentration (mg/L, mean ± SEM) of patients in group A (Kan Jang) and group B (placebo) over the time from Day 1 to Day 14. The changes from the baseline within groups A and B over time were significant (p<0.0001); between-groups comparison of the changes of blood serum C-reactive protein concentration from the baseline over time shows no interaction (p=0.8202), and no significant difference (p =0.3920) between groups A and B. The Kan Jang treatment has no statistically significant effect on Blood serum C-reactive protein concentration compared to placebo.

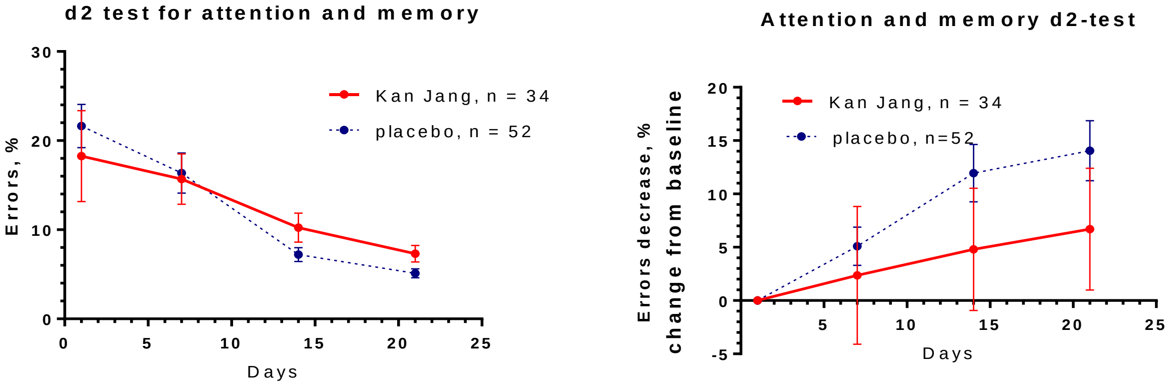

Figure S6. (a) – Errors, % in d2 test for attention and memory (mean ± SEM) of patients in group A (Kan Jang) and group B (placebo) over the time from Day 1 to Day 21. The changes from the baseline within groups A and B over time were significant (*p*<0.0001; calculated by Repeated measures ANOVA); two-way ANOVA analysis showed no significant interaction between groups over time; *p*= 0.6068. The number of patients included in the analysis is shown in Tables 11 and 12; (b) Between-groups comparison of the changes of Errors, % from the baseline over time shows no interaction (p= 0.6471), and no significant difference (p=0.0798) between groups A and B. The Kan Jang treatment has no statistically significant effect on cognitive functions of patients compared to placebo.

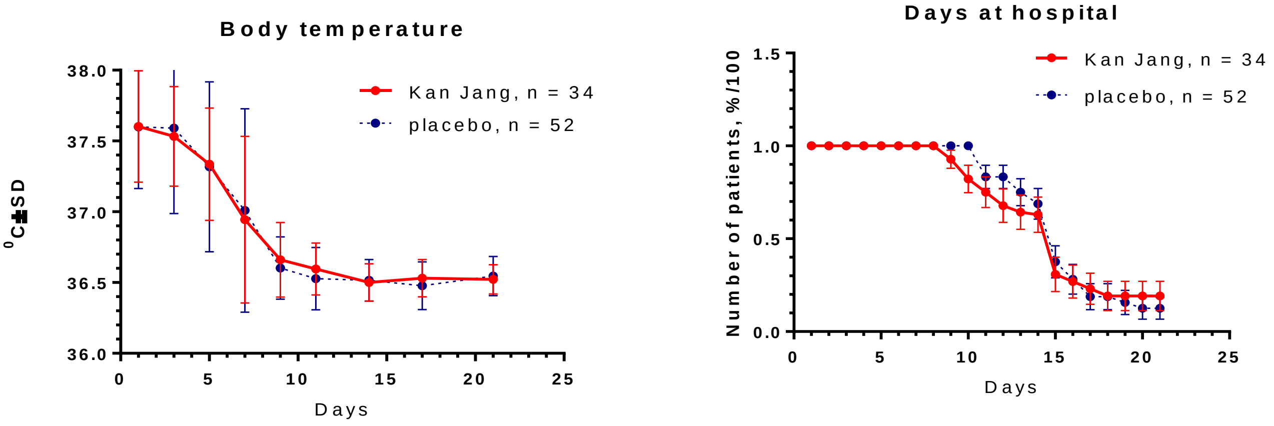

Figure S7. (a) – Body temperature C^{O} (C^{O}, mean ± SEM) of patients in group A (Kan Jang) and group B (placebo) over the time from Day 1 to Day 21. The changes from the baseline within groups A and B over time were significant (p<0.0001; calculated by Repeated measures ANOVA); two-way ANOVA showed no significant interaction (p= 9815) and no significant difference between groups over time, p= 0.8897. (b) Between-groups comparison of the days at hospital from the baseline over time shows no interaction (p= 0.7475), and no significant difference (p = 0.1251) between groups A and B. However, Kan Jang treatment decreases the number of patients in hospital from day 9 to day 14 compared to placebo.

Data Sets Analyzed

All enrolled and randomly allocated to treatment were included in the intention to treat analysis. Efficacy subset analysis per-protocol (P.P.) was performed for the subset of patients with Mild Covid Symptoms and the baseline (visit 1) and completed the study therapy. A per-protocol (P.P.) analysis aims to identify a treatment effect on the symptoms. Therefore, some patients (from the complete analysis set) need to be excluded from the population used for the P.P. analysis (P.P. population), Table S1.

Table S1 Disposition of patients (Source Data: Supplement 1)

	Group A	Group B	Total
Enrolled	34 (100%)	52 (100%)	86 (100%)
Received at least one dose	34 (100%)	52 (100%)	86 (100%)
Completed study treatments	27 (79%)	31 (60%)	58 (67%)
Completed first visit	34 (100%)	52 (100%)	86 (100%)
Completed all visits	27 (79%)	31 (60%)	58 (67%)
Included in ITT analysis:	34 (100%)	52 (100%)	86 (100%)

• Fatigue	34 (100%)	52 (100%)	86 (100%)
• Headache	31 (90%)	42 (82%)	86 (86%)
• Sore throat	18 (38%)	18 (38%)	38 (38%)
• Cough	15 (52%)	**18** (44%)	33 (48%)
• Muscle pain	11 (66%)	**22** (74%)	33 (70%)
• Runny nose	5 (64%)	**6** (54%)	11 (59%)
• Loss of smell	2 (92%)	**5** (88%)	7 (90%)
• Loss of taste	0 (0%)	**0** (82%)	0 (82%)
• Physical activity	34 (100%)	52 (100%)	86 (100%)
• Physical activity (daily walk)	34 (100%)	52 (100%)	86 (100%)
• Decreased attention (d2-test)	34 (100%)	52 (100%)	86 (100%)
• URTI	34 (100%)	52 (100%)	86 (100%)
• QOL	34 (100%)	52 (100%)	86 (100%)
• Blood serum cytokines IL-6	34 (100%)	52 (100%)	86 (100%)
• D-Dimer	34 (100%)	52 (100%)	86 (100%)
• C-reactive protein	34 (100%)	52 (100%)	86 (100%)
• Body temperature	34 (100%)	52 (100%)	86 (100%)
• Viral load, SARS-Cov2	34 (100%)	52 (100%)	86 (100%)
Included in analysis per-protocol, P.P:			
• Fatigue	34 (100%)	52 (100%)	86 (100%)
• Headache	31 (90%)	42 (82%)	86 (86%)
• Sore throat	18 (38%)	18 (38%)	38 (38%)
• Cough	15 (52%)	**18** (44%)	33 (48%)
• Muscle pain	11 (66%)	**22** (74%)	33 (70%)
• Runny nose	5 (64%)	**6** (54%)	11 (59%)
• Loss of smell	2 (92%)	**5** (88%)	7 (90%)
• Loss of taste	0 (0%)	**0** (82%)	0 (82%)
• Physical activity	27 (100%)	32 (100%)	59 (100%)
• Physical activity (daily walk)	27 (100%)	32 (100%)	59 (100%)
• Decreased attention (d2-test)	27 (100%)	32 (100%)	59 (100%)
• URTI	27 (100%)	32 (100%)	59 (100%)
• QOL	27 (100%)	32 (100%)	59 (100%)
• Blood serum cytokines IL-6	27 (100%)	32 (100%)	59 (100%)
• D-Dimer	27 (100%)	32 (100%)	59 (100%)
• C-reactive protein	27 (100%)	32 (100%)	59 (100%)
• Body temperature	27 (100%)	32 (100%)	59 (100%)
• Viral load, SARS-Cov2	27 (100%)	32 (100%)	59 (100%)
