# Supplementary material for: Efficacy of Kan Jang® in Patients with Mild COVID-19: Interim Analysis of a Randomized, Quadruple-Blind, Placebo-Controlled Trial
Source: Pharmaceuticals (Basel). 2022 Aug 17;15(8):1013. doi: 10.3390/ph15081013 (PMC9415141; doi:10.3390/ph15081013)
Supplement: Supplementary file 1 [file pharmaceuticals-15-01013-s001.zip › Supplement S4 CRF-Kan Jang in COVID-19 and ammendment 2022-05-06.pdf]

CASE REPORT FORM

Effect of Kan-Jang® supplementation in Patients Diagnosed With COVID-19: A Randomized, Quadruple-blind, Placebo-controlled Trial.

SPONSOR: Swedish Herbal Institute AB, Sweden

STUDY NO. 2021-02

Treatment random No ______ ______

Participant's ID/Subject initials: ………………………………………………………….

Investigator signature: ______________________

Tbilisi, Georgia

2022

TITLE PAGE

**Protocol Title**	Effect of Kan-Jang® supplementation in Patients Diagnosed With COVID-19: A Randomized, Quadruple-blind, Placebo-controlled Trial
**Protocol No:**	SHIRD-2021-2
**Sponsor**	Karl Georg Wikman,<br>Founder & President<br>Swedish Herbal Institute AB.<br>21 Kövlingevägen,<br>Vallberga, 31250, Halland, Sweden<br>Phone: +46-702733753<br>Email: karl.georg.wikman@gmail.com<br>https://www.shi.se/
**Clinical Trial Site**	The First University Clinic of Tbilisi State Medical University<br>Address: 4 Gudamakari St., 0141 Tbilisi, Georgia<br>https://vipmed.ge/levan-ratiani-en/
**Principal Investigator**	Name Prof. Levan Ratiani - MD, PhD,<br><br>Signature: ________________________________<br><br>Date : ________________________________

CONFIDENTIALITY STATEMENT

This confidential document is the property of Swedish Herbal Institute AB.
No unpublished information contained herein may be disclosed without prior written approval from Swedish Herbal Institute AB, Sweden
Access to this document must be restricted to the relevant parties only

GUIDELINES

(FOR RECORDING DATA AND COMPLETION OF CASE REPORT FORMS)

1. Make all entries in **BLACK INK** to maximize legibility and facilitate copying.
2. Write legibly and neatly in the space provided.

   e.g. : 0 1 or use tick mark in the boxes provided.

3. Use acceptable and uniform medical terminology throughout the CRF.
4. All comments should be brief, concise, clear, and confined to "comment" sections of the CRF. Write comments only if they apply to the study and the topic.
5. For recording subject initials, the first letter of the patient name followed by the first letter of the middle and last name. If there is no middle name the first two letters of the first name and the first letter of the last name should be used. In case of similar initials, the first and last letters of the first name and the first letter of the last name should be used. All initials should follow the same format. If there are more similar initials you can select any letter from the name that will create a unique initial.

   For e.g. :

   Peter Heck John Initial: P H J

   Paul John Initial : PAJ

   Pasteur Jennings Initial : PRJ

6. In case of correction DO NOT write over data, erase data, or use correction fluid. Do a straight single cut and write the data nearby with your initial and date of correction. Even if there is only a letter or number error you will have to cut the whole word or number and write above.

   R K N PK 03/12/2012 81.50 .50 PK 03/12/2012

   e.g. : ~~R N K~~ correct ~~8 1 . 0 5~~ correct 8 1. ~~0 5~~ wrong

7. Complete all blanks. If data are missing, use one of the following conventions:

   NA (Not Applicable) / ND (Not Done) / UNK (Unknown)

8. Verify all UNITS are recorded as specified in the CRF.

9. TIME to be recorded in a 24 hour clock HH / MM format only.

   e.g.: 1 3. 5 0 for 1. 50 p.m.

10. Enter all dates only in DD/ MMM / YY format.

    e.g.: 10/ OCT / 17

11. Use a BLACK CARBON SHEET in between two similar CRF pages to obtain the imprint on the copy page which will be considered the 'Duplicate Copy' of CRF that will remain at the site. The 'Original Copy' will be retrieved by the CRO for Data Entry purposes and documentation thereafter.

12. The investigator must review and sign off each CRF (at the end of each visit) as a confirmation of his/her review

Subject Initials	[ ][ ][ ]	Treatment random no	[ ][ ]	Date:	[ ][ ] / [ ][ ] / [ ][ ]
					Month Day Year

Demographics

Subject First University Clinic Medical Record Number*: [ ][ ][ ][ ][ ][ ][ ]

**First Name*:**
**Middle Name (or initial):**
**Last Name*:**

Birthdate*: [ ][ ] / [ ][ ] / [ ][ ][ ][ ]
Month Day Year

Contact Information:

**Address:**		**Unit #:**
**City:**	**State:**	**Zip:**
**Phone Number:** [ ]<br>☐ Home ☐ Work<br>☐ Cell ☐ Other	**Alternate Phone Number:** [ ]<br>☐ Home ☐ Work<br>☐ Cell ☐ Other	**Email address:**
**Preferred method of contact:**		

Emergency Contact:

**Name:**		
**Address:**		**Unit #:**
**City:**	**State:**	**Zip:**
**Phone Number:** [ ]<br>☐ Home ☐ Work<br>☐ Cell ☐ Other	**Alternate Phone Number:** [ ]<br>☐ Home ☐ Work<br>☐ Cell ☐ Other	**Email address:**
**Preferred method of contact:**		

Form Completed By: ______________________________ **Date:** ______________

Site PI Signature: ______________________________ Date: ______________

Dr. Levan Ratiani, PhD, MD

,

Subject Initials ☐☐☐ **Treatment random no** ☐☐ **Date:** ☐☐/☐☐/☐☐

Informed Consent Authorization Obtained

☐ Consent Refused

Date Signed: ☐☐/☐☐/☐☐ **Time:** ☐☐:☐☐ (using 24 hour format)

Document(s) signed	Version Date	Approval Date
Consent Form	2021-03-23	2021-03-23

Consent Form, and related study documents, was thoroughly reviewed with the subject.
☐ Yes ☐ No

Subject had sufficient time to review the documents and ask questions.
☐ Yes ☐ No

Informed consent/Authorization obtained prior to any study related procedures.
☐ Yes ☐ No

A copy of the signed documents have been given to the subject.
☐ Yes ☐ No

Name of person that obtained consent: ☐

Comments: ☐

Informed Consent Refused

Time: ☐☐:☐☐ (using 24 hour format) (e.g. hh:mm) ☐ *Not Applicable*

Comments: ☐

Form Completed by: ________________________________ Date: ______________

Site PI Signature: ________________________________ Date: ______________

VISIT 1 - SCREENING

**Subject Screening No: __ __ __ __ __**	**Subject Initials : __ __ __**
**MEDICATION HISTORY**	
Has the patient taken any medication for any illness (past 6 MMMs)<br>☐ Yes ☐ No	Date of Entry : __ __ / __ __ / __ __<br>DD MM YY
Medication (Generic Name):<br>Dosage: __ __ __ __ mg Frequency: __ times daily<br>Indication:	Start Date : __ __ / __ __ / __ __<br>DD MM YY<br>End Date : __ __ / __ __ / __ __<br>DD MM YY<br>☐ Ongoing
Medication (Generic Name):<br>Dosage: __ __ __ __ mg Frequency: __ times daily<br>Indication:	Start Date : __ __ / __ __ / __ __<br>DD MM YY<br>End Date : __ __ / __ __ / __ __<br>DD MM YY<br>☐ Ongoing
Medication (Generic Name):<br>Dosage: __ __ __ __ mg Frequency: __ times daily<br>Indication:	Start Date : __ __ / __ __ / __ __<br>DD MM YY<br>End Date : __ __ / __ __ / __ __<br>DD MM YY<br>☐ Ongoing
Medication (Generic Name):<br>Dosage: __ __ __ __ mg Frequency: __ times daily<br>Indication:	Start Date : __ __ / __ __ / __ __<br>DD MM YY<br>End Date : __ __ / __ __ / __ __<br>DD MM YY<br>☐ Ongoing

Form Completed By: ____ **Date:** _______________

Site PI Signature: ____ Date: _______________

VISIT 1 - SCREENING

**Subject Screening No: __ __ __ __ __**	**Subject Initials : __ __ __**

PHYSICAL EXAMINATION

Physical Exam Performed: ☐ Yes ☐ No	Date: __ __ / __ __ / __ __ (DD / MM / YY)

System	Normal	Abnormal*	Not done	* If Abnormal, comment briefly and indicate CS/ NCS
General Appearance	☐	☐	☐	
Skin	☐	☐	☐	
Eyes, Ears, Nose & Throat	☐	☐	☐	
Head, Neck & Thyroid	☐	☐	☐	
Cardiovascular	☐	☐	☐	
Respiratory	☐	☐	☐	
Abdomen	☐	☐	☐	
Extremities	☐	☐	☐	
Genitalia	☐	☐	☐	
Anorectal	☐	☐	☐	
Lymph Nodes	☐	☐	☐	
Muscular-Skeletal	☐	☐	☐	
Neurological	☐	☐	☐	
Others (please specify)	☐	☐	☐	

Form Completed By: ____ **Date:** _______________

Site PI Signature: ____ Date: _______________

VISIT 1 - SCREENING.

**Subject Screening No: __ __ __ __ __**	**Subject Initials: __ __ __**
**CONCOMITANT MEDICATION**	
At the time of screening is the patient taking any medications.<br>☐ Yes ☐ No	Date of Entry: __ __ / __ __ / __ __<br>DD MM YY
Medication (Generic Name):<br>Dosage: __ __ __ __ mg Frequency: __ times daily<br>Indication:	Start Date : __ __ / __ __ / __ __<br>DD MM YY<br>End Date : __ __ / __ __ / __ __<br>DD MM YY<br>☐ Ongoing
Medication (Generic Name):<br>Dosage: __ __ __ __ mg Frequency: __ times daily<br>Indication:	Start Date : __ __ / __ __ / __ __<br>DD MM YY<br>End Date : __ __ / __ __ / __ __<br>DD MM YY<br>☐ Ongoing
Medication (Generic Name):<br>Dosage: __ __ __ __ mg Frequency: __ times daily<br>Indication:	Start Date : __ __ / __ __ / __ __<br>DD MM YY<br>End Date : __ __ / __ __ / __ __<br>DD MM YY<br>☐ Ongoing
Medication (Generic Name):<br>Dosage: __ __ __ __ mg Frequency: __ times daily<br>Indication:	Start Date : __ __ / __ __ / __ __<br>DD MM YY<br>End Date : __ __ / __ __ / __ __<br>DD MM YY<br>☐ Ongoing

Form Completed By: ____ **Date:** _______________

Site PI Signature: ____ Date: ______________

,

VISIT 1 - SCREENING

**Subject Screening No: __ __ __ __ __**					**Subject Initials : __ __ __**
**LABORATORY INVESTIGATION**					
Was blood collected in this visit?<br>☐ Yes ☐ No Quantity : __ __ mL<br>Time : __ __ : __ __<br>Hour Min					Date of Collection : __ __ / __ __ / __ __<br>DD MM YY<br>Date of Report : __ __ / __ __ / __ __<br>DD MM YY

Visits

**Parameter**	**Normal Reference Range**	**Visit 1 Day 1 Baseline**	**Day 3**	**Visit 2 Day 7**	**Visit 3 Day 14**	**Visit 4 Day 21 Follow up**
SARS-Cov2 PCR test						
Blood serum IL-6, pg/ml						
D-Dimer						
C-reactive protein						

Liver and Renal Function Tests

AST(aspartate amino transferase, SGOT)	☐	☐	☐
ALT (alanine aminotransferase, SGPT)	☐	☐	☐

Form Completed By: ____ **Date:** ______________

Site PI Signature: ____ Date: ______________

,

VISIT 1 - SCREENING

**Subject Screening No: __ __ __ __ __**			**Subject Initials : __ __ __**		
**Complete Blood Count**					
**Parameter**	**Value**	**Reference Range**	**CS**	**NCS**	**ND**
Hemoglobin			☐	☐	☐
RBC			☐	☐	☐
Hematocrit			☐	☐	☐
Total Leukocyte count			☐	☐	☐
Total Lymphocyte count			☐	☐	☐
Absolute Neutrophil count			☐	☐	☐
Eosinophil count			☐	☐	☐
Basophil Count			☐	☐	☐
Monocyte count			☐	☐	☐
Platelet Count			☐	☐	☐
ESR			☐	☐	☐

Form Completed By: ____ **Date:** _________________

Site PI Signature: ____ Date: _______________

VISIT 1 - SCREENING

Subject Initials ☐☐☐ **Treatment random no** ☐☐ **Date:** ☐☐/☐☐/☐☐

Month Day Year

Eligibility Criteria

Inclusion Criteria

Patients who meet *all* of the following criteria are eligible for enrollment as study participants:

	Yes	No
COVID-19 patients with confirmed diagnosis based on positive SARS-Cov-2 test (in the last 7 days) and at least three of COVID-19 symptoms listed below:		
• fatigue (asthenia)		
• headache,		
• loss of smell		
• gustatory dysfunction		
• rhinorrhea,		
• nasal congestions		
• cough		
• sore throat,		
• myalgia		
• fever (high temperature)		
COVID-19 patient in stable moderate condition (i.e., not requiring Intensive Care Unit (ICU) admission).		
Subjects must be under observation or admitted to a controlled facility or hospital (home quarantine is not sufficient).		
Able to take medication alone;		
Able to understand and provide signed informed consent;		
Able to participate in a 3-week study.		
18 Years and older (Adult, Older Adult)		
**The patient meets the inclusion criteria**		

Form Completed By: ______________________________ **Date:** ______________

Site PI Signature: ______________________________ Date: ______________

VISIT 1 - SCREENING

Subject Initials | | | | **Treatment random no** | | | **Date:** | | |/| | |/| | |

Month Day Year

Eligibility Criteria

Exclusion Criteria

Patients who meet *any* of these criteria are *not* eligible for enrollment as study participants:

	Yes	No
Pulmonary diseases		
Chronic pulmonary diseases		
Chronic rhinosinusitis		
Patient admitted already under invasive mechanical ventilation;		
Patient admitted with severe acute respiratory syndrome and diagnosed with an etiologic agent other than Covid 19;		
Renal failure requiring dialysis or creatinine ≥ 2.0mg/dl;		
Tube feeding or parenteral nutrition.		
Respiratory decompensation requiring mechanical ventilation.		
Uncontrolled diabetes type 2.		
Hypertension stage 3,		
Autoimmune disease.		
Pregnant or lactating women.		
Taking antibiotics for a reason other than COVID-19 at enrollment.		
Has a chronically weakened immune system (AIDS, lymphoma, chemo-radio- corticosteroid therapy, immunosuppressive pathology);		
Was treated with chemo-radio-corticosteroid therapy in the last 6 months.		
Has active cancer.		
Taking immunosuppressive drugs (e.g. anti-rejection treatment after organ transplant);		
Already participating in another clinical trial;		
Has any other condition that would prevent safe participation in the study		
**The patient meets the exclusion criteria**		

Declaration by the principal investigator: I have verified the data entered in the Screening Visit for this subject and certify that it is accurate and complete.

Form Completed by: ____ Date: _______________

Site PI Signature: ____ Date: _______________

Subject Initials ☐☐☐ **Treatment random no** ☐☐ **Date:** ☐☐/☐☐/☐☐

Month Day Year

START OF SYMPTOMS: (When did you first experience the first signs of COVID symptoms?)

- ☐ 0-1 days ago
- ☐ 1-2 days ago
- ☐ 2-3 days ago
- ☐ 4-7 days ago

Form Completed By: ______________________________ **Date:** ______________

Site PI Signature: ______________________________ Date: ______________

Subject Initials ☐☐☐ **Treatment random no** ☐☐ **Date:** ☐☐ / ☐☐ / ☐☐

VISIT 1 - SCREENING

**Subject Random No: __ __ __ __ __**			**Subject Initials : __ __ __**		
**TREATMENT ALLOCATION**					
Was the patient is allocated to a treatment arm?<br>Yes ☐ No ☐		Date: __ __ / __ __ / __ __<br>DD MM YY			
Randomization number given:<br>___ ___ ___ ___	Bottle No:<br>___	No: of capsules<br>___ ___ ___	Batch No:<br>___ ___ ___ ___ ___/___ ___		
**CONCOMITANT MEDICATION**				**Yes**	**No**
Are there any changes in concomitant medication since the last visit?<br>If yes, record on the Concomitant Medication Log.				☐	☐
**INVESTIGATOR VERIFICATION**					
I have verified the data entered in Visit 3 for this subject and certify that it is accurate and complete.					

Form Completed By: ________________________________ **Date:** ________________

Site PI Signature: ________________________________ Date: ________________

Day 1: Baseline

The URTI symptom score is calculated by summing of 10 URTI symptom questions. ________________
The QOL score is calculated by summing of 9 QOL questions during the study. _____________________
The total URTI symptom is an overall condition expressed by summing of URTI symptom and QOL scores:___________

Wisconsin Upper Respiratory Symptom Survey – 21 --- *Daily Symptom Report*

Day:	*Date:*	*Time:*	*ID:*

Please fill in one circle for each of the following items:

	Not sick	Very mildly		Mildly		Moderately		Severely
	**0**	**1**	**2**	**3**	**4**	**5**	**6**	**7**
How sick do you feel **today**?	O	O	O	O	O	O	O	O

Please rate the **average severity of your cold symptoms over the last 24 hours** for each symptom:

**URTI symptom**	**Do not have this symptom**	Very mild		Mild		Moderate		Severe
	**0**	**1**	**2**	**3**	**4**	**5**	**6**	**7**
Runny nose	O	O	O	O	O	O	O	O
Plugged nose	O	O	O	O	O	O	O	O
Sneezing	O	O	O	O	O	O	O	O
Sore throat	O	O	O	O	O	O	O	O
Scratchy throat	O	O	O	O	O	O	O	O
Cough	O	O	O	O	O	O	O	O
Hoarseness	O	O	O	O	O	O	O	O
Head congestion	O	O	O	O	O	O	O	O
Chest congestion	O	O	O	O	O	O	O	O
Feeling tired	O	O	O	O	O	O	O	O

Over the last 24 hours, how much has your cold interfered with your ability to:

**QOL measures**	**Not at all**	Very mildly		Mildly		Moderately		Severely
	**0**	**1**	**2**	**3**	**4**	**5**	**6**	**7**
Think clearly	O	O	O	O	O	O	O	O
Sleep well	O	O	O	O	O	O	O	O
Breathe easily	O	O	O	O	O	O	O	O
Walk, climb stairs, exercise	O	O	O	O	O	O	O	O
Accomplish daily activities	O	O	O	O	O	O	O	O
Work outside the home	O	O	O	O	O	O	O	O
Work inside the home	O	O	O	O	O	O	O	O
Interact with others	O	O	O	O	O	O	O	O
Live your personal life	O	O	O	O	O	O	O	O

Compared to yesterday, I feel that my cold is…

Very much better	Somewhat better	A little better	The same	A little worse	Somewhat worse	Very much worse
O	O	O	O	O	O	O

Day 3

The URTI symptom score is calculated by summing of 10 URTI symptom questions. ________________

The QOL score is calculated by summing of 9 QOL questions during the study. ____________________

The total URTI symptom is an overall condition expressed by summing of URTI symptom and QOL scores:___________

Wisconsin Upper Respiratory Symptom Survey – 21 --- *Daily Symptom Report*

Day:	*Date:*	*Time:*	*ID:*

Please fill in one circle for each of the following items:

	**Not sick**	Very mildly		Mildly		Moderately		Severely
	**0**	**1**	**2**	**3**	**4**	**5**	**6**	**7**
How sick do you feel **today**?	○	○	○	○	○	○	○	○

Please rate the **average severity of your cold symptoms over the last 24 hours** for each symptom:

URTI symptom	**Do not have this symptom**	Very mild		Mild		Moderate		Severe
	**0**	**1**	**2**	**3**	**4**	**5**	**6**	**7**
Runny nose	○	○	○	○	○	○	○	○
Plugged nose	○	○	○	○	○	○	○	○
Sneezing	○	○	○	○	○	○	○	○
Sore throat	○	○	○	○	○	○	○	○
Scratchy throat	○	○	○	○	○	○	○	○
Cough	○	○	○	○	○	○	○	○
Hoarseness	○	○	○	○	○	○	○	○
Head congestion	○	○	○	○	○	○	○	○
Chest congestion	○	○	○	○	○	○	○	○
Feeling tired	○	○	○	○	○	○	○	○

Over the last 24 hours, how much has your cold interfered with your ability to:

QOL measures	**Not at all**	Very mildly		Mildly		Moderately		Severely
	**0**	**1**	**2**	**3**	**4**	**5**	**6**	**7**
Think clearly	○	○	○	○	○	○	○	○
Sleep well	○	○	○	○	○	○	○	○
Breathe easily	○	○	○	○	○	○	○	○
Walk, climb stairs, exercise	○	○	○	○	○	○	○	○
Accomplish daily activities	○	○	○	○	○	○	○	○
Work outside the home	○	○	○	○	○	○	○	○
Work inside the home	○	○	○	○	○	○	○	○
Interact with others	○	○	○	○	○	○	○	○
Live your personal life	○	○	○	○	○	○	○	○

Compared to yesterday, I feel that my cold is…

Very much better	Somewhat better	A little better	The same	A little worse	Somewhat worse	Very much worse
○	○	○	○	○	○	○

Day 7

The URTI symptom score is calculated by summing of 10 URTI symptom questions. ________________
The QOL score is calculated by summing of 9 QOL questions during the study. _____________________
The total URTI symptom is an overall condition expressed by summing of URTI symptom and QOL scores:___________

Wisconsin Upper Respiratory Symptom Survey – 21 --- *Daily Symptom Report*

Day:	*Date:*	*Time:*	*ID:*

Please fill in one circle for each of the following items:

	Not sick	Very mildly		Mildly		Moderately		Severely
	**0**	**1**	**2**	**3**	**4**	**5**	**6**	**7**
How sick do you feel **today**?	O	O	O	O	O	O	O	O

Please rate the **average severity of your cold symptoms over the last 24 hours** for each symptom:

URTI symptom	Do not have this symptom	Very mild		Mild		Moderate		Severe
	**0**	**1**	**2**	**3**	**4**	**5**	**6**	**7**
Runny nose	O	O	O	O	O	O	O	O
Plugged nose	O	O	O	O	O	O	O	O
Sneezing	O	O	O	O	O	O	O	O
Sore throat	O	O	O	O	O	O	O	O
Scratchy throat	O	O	O	O	O	O	O	O
Cough	O	O	O	O	O	O	O	O
Hoarseness	O	O	O	O	O	O	O	O
Head congestion	O	O	O	O	O	O	O	O
Chest congestion	O	O	O	O	O	O	O	O
Feeling tired	O	O	O	O	O	O	O	O

Over the last 24 hours, how much has your cold interfered with your ability to:

QOL measures	Not at all	Very mildly		Mildly		Moderately		Severely
	**0**	**1**	**2**	**3**	**4**	**5**	**6**	**7**
Think clearly	O	O	O	O	O	O	O	O
Sleep well	O	O	O	O	O	O	O	O
Breathe easily	O	O	O	O	O	O	O	O
Walk, climb stairs, exercise	O	O	O	O	O	O	O	O
Accomplish daily activities	O	O	O	O	O	O	O	O
Work outside the home	O	O	O	O	O	O	O	O
Work inside the home	O	O	O	O	O	O	O	O
Interact with others	O	O	O	O	O	O	O	O
Live your personal life	O	O	O	O	O	O	O	O

Compared to yesterday, I feel that my cold is…

Very much better	Somewhat better	A little better	The same	A little worse	Somewhat worse	Very much worse
O	O	O	O	O	O	O

VISIT 3

Day 14

The URTI symptom score is calculated by summing of 10 URTI symptom questions. ________________
The QOL score is calculated by summing of 9 QOL questions during the study. ____________________
The total URTI symptom is an overall condition expressed by summing of URTI symptom and QOL scores:___________

Wisconsin Upper Respiratory Symptom Survey – 21 --- *Daily Symptom Report*

Day:	*Date:*	*Time:*	*ID:*

Please fill in one circle for each of the following items:

	Not sick 0	Very mildly 1	2	Mildly 3	4	Moderately 5	6	Severely 7
How sick do you feel **today**?	O	O	O	O	O	O	O	O

Please rate the **average severity of your cold symptoms over the last 24 hours** for each symptom:

URTI symptom	Do not have this symptom 0	Very mild 1	2	Mild 3	4	Moderate 5	6	Severe 7
Runny nose	O	O	O	O	O	O	O	O
Plugged nose	O	O	O	O	O	O	O	O
Sneezing	O	O	O	O	O	O	O	O
Sore throat	O	O	O	O	O	O	O	O
Scratchy throat	O	O	O	O	O	O	O	O
Cough	O	O	O	O	O	O	O	O
Hoarseness	O	O	O	O	O	O	O	O
Head congestion	O	O	O	O	O	O	O	O
Chest congestion	O	O	O	O	O	O	O	O
Feeling tired	O	O	O	O	O	O	O	O

Over the last 24 hours, how much has your cold interfered with your ability to:

QOL measures	Not at all 0	Very mildly 1	2	Mildly 3	4	Moderately 5	6	Severely 7
Think clearly	O	O	O	O	O	O	O	O
Sleep well	O	O	O	O	O	O	O	O
Breathe easily	O	O	O	O	O	O	O	O
Walk, climb stairs, exercise	O	O	O	O	O	O	O	O
Accomplish daily activities	O	O	O	O	O	O	O	O
Work outside the home	O	O	O	O	O	O	O	O
Work inside the home	O	O	O	O	O	O	O	O
Interact with others	O	O	O	O	O	O	O	O
Live your personal life	O	O	O	O	O	O	O	O

Compared to yesterday, I feel that my cold is…

Very much better	Somewhat better	A little better	The same	A little worse	Somewhat worse	Very much worse
O	O	O	O	O	O	O

VISIT 1

Day 1: Baseline

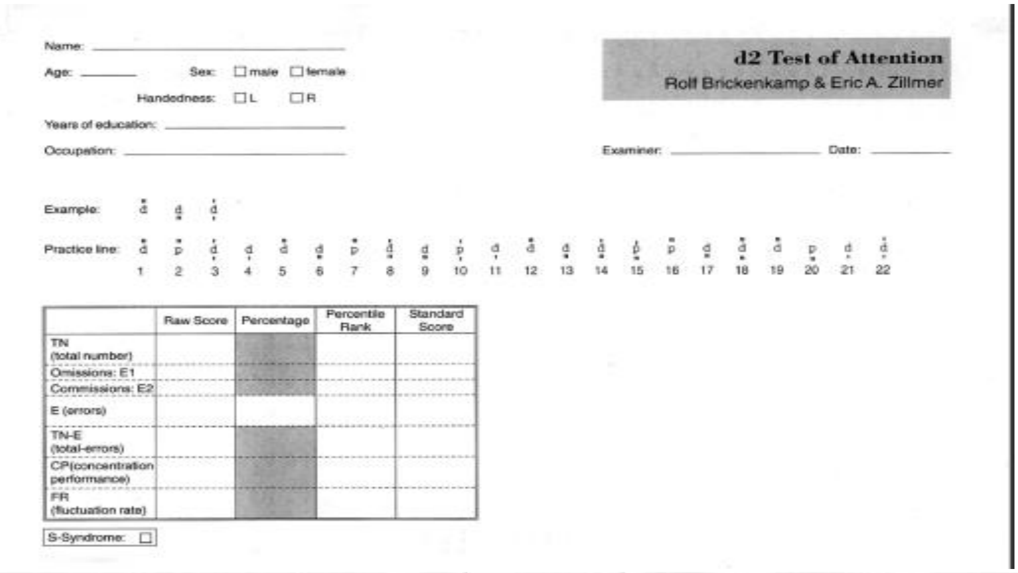

Name: ______________

Age: ______ Sex: ☐ male ☐ female

Handedness: ☐ L ☐ R

Years of education: ______________

Occupation: ______________

d2 Test of Attention
Rolf Brickenkamp & Eric A. Zillmer

Examiner: ______________ Date: ________

Example:

Practice line: 1 2 3 4 5 6 7 8 9 10 11 12 13 14 15 16 17 18 19 20 21 22

	Raw Score	Percentage	Percentile Rank	Standard Score
TN (total number)				
Omissions: E1				
Commissions: E2				
E (errors)				
TN-E (total-errors)				
CP(concentration performance)				
FR (fluctuation rate)				

S-Syndrome: ☐

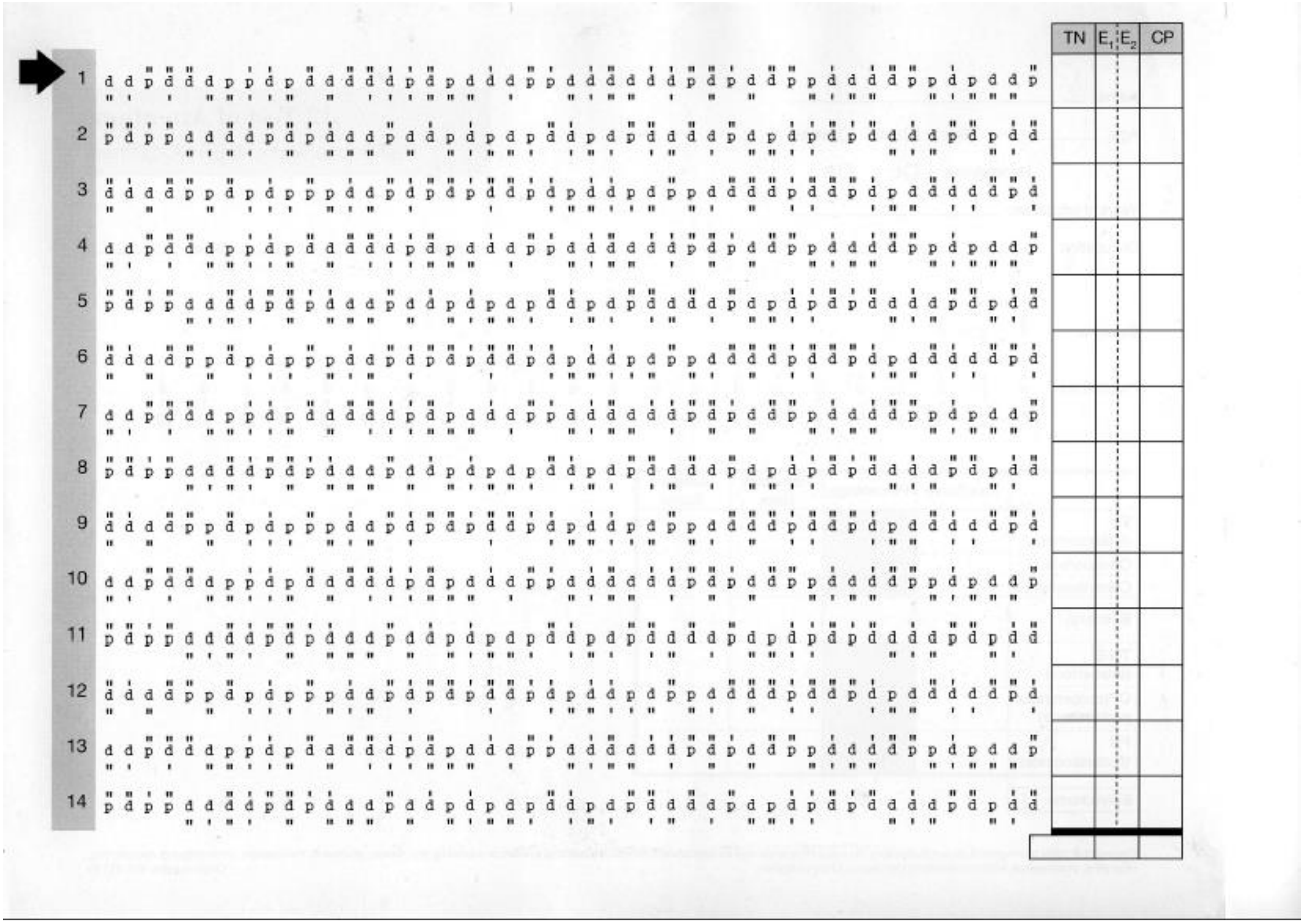

Time of completed _____ h ______ min

VISIT 2

Day 7

Name: ______________________

Age: ________ Sex: ☐ male ☐ female

Handedness: ☐ L ☐ R

Years of education: ______________________

Occupation: ______________________

d2 Test of Attention
Rolf Brickenkamp & Eric A. Zillmer

Examiner: ______________________ Date: ____________

Example:

Practice line: 1 2 3 4 5 6 7 8 9 10 11 12 13 14 15 16 17 18 19 20 21 22

	Raw Score	Percentage	Percentile Rank	Standard Score
TN (total number)				
Omissions: E1				
Commissions: E2				
E (errors)				
TN-E (total-errors)				
CP(concentration performance)				
FR (fluctuation rate)				

S-Syndrome: ☐

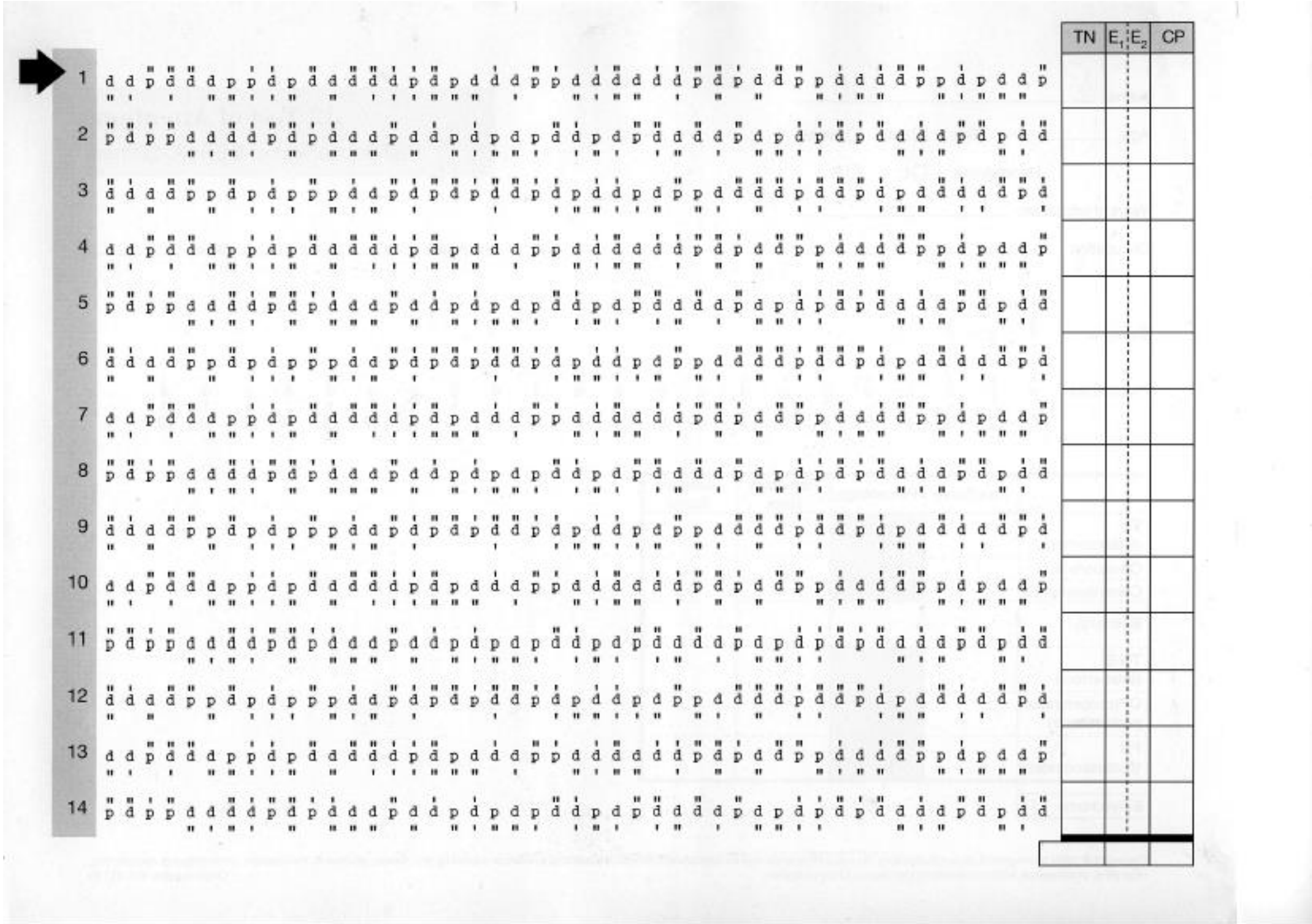

Time of completed _____ h ______ min

Day 14

d2 Test of Attention
Rolf Brickenkamp & Eric A. Zillmer

Name: ____________________

Age: ________ Sex: ☐ male ☐ female

Handedness: ☐ L ☐ R

Years of education: ____________________

Occupation: ____________________

Examiner: ______________ Date: __________

Example:

Practice line:

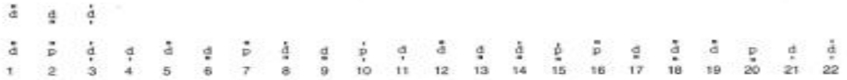

	Raw Score	Percentage	Percentile Rank	Standard Score
TN (total number)				
Omissions: E1				
Commissions: E2				
E (errors)				
TN-E (total-errors)				
CP(concentration performance)				
FR (fluctuation rate)				

S-Syndrome: ☐

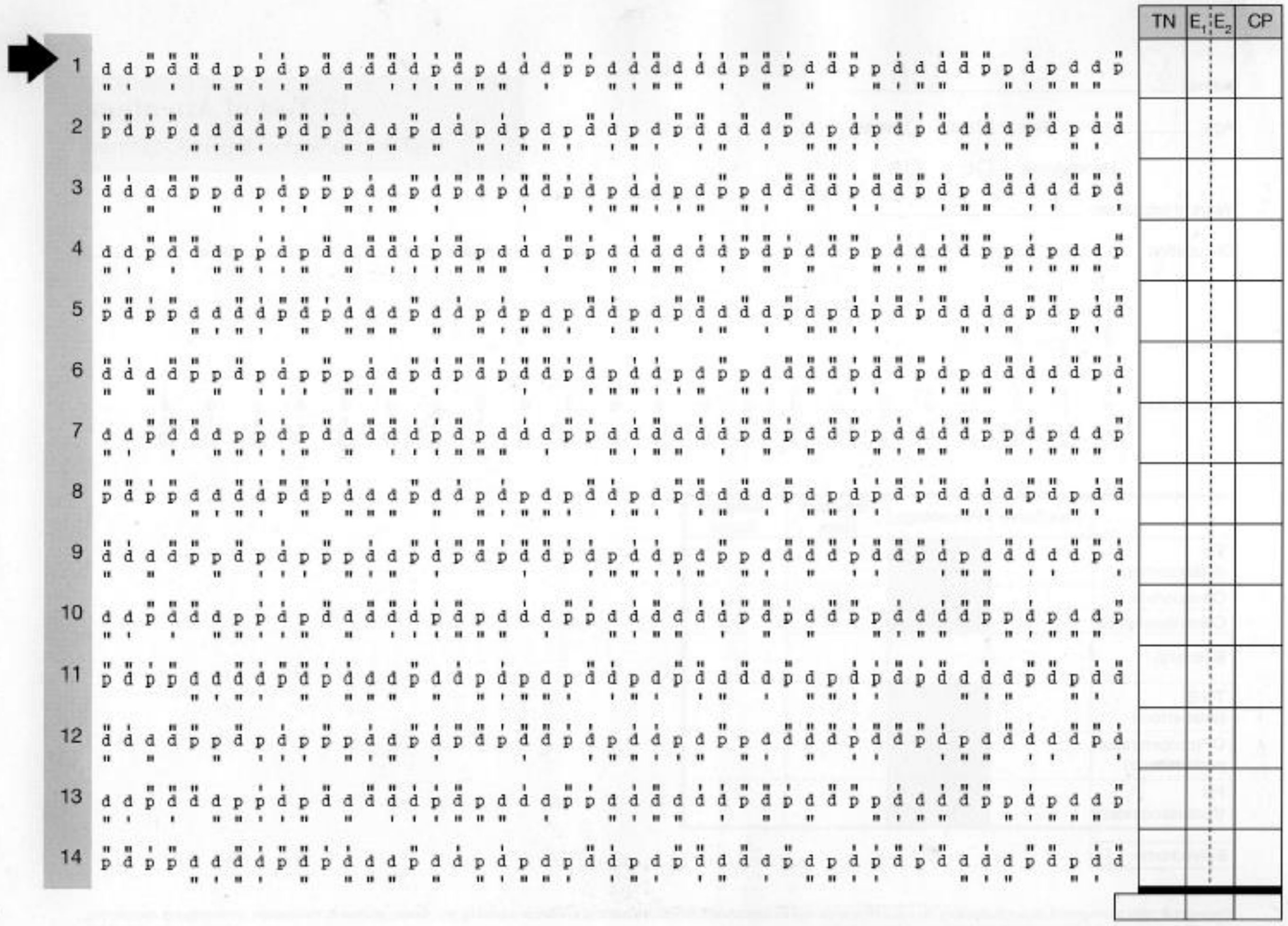

Time of completed _____ h ______ min

Day 21

Name: ____________________

Age: ________ Sex: ☐ male ☐ female

Handedness: ☐ L ☐ R

Years of education: ____________________

Occupation: ____________________

d2 Test of Attention
Rolf Brickenkamp & Eric A. Zillmer

Examiner: ____________ Date: ________

Example:

Practice line:

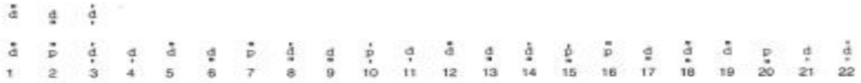

	Raw Score	Percentage	Percentile Rank	Standard Score
TN (total number)				
Omissions: E1				
Commissions: E2				
E (errors)				
TN-E (total-errors)				
CP(concentration performance)				
FR (fluctuation rate)				

S-Syndrome: ☐

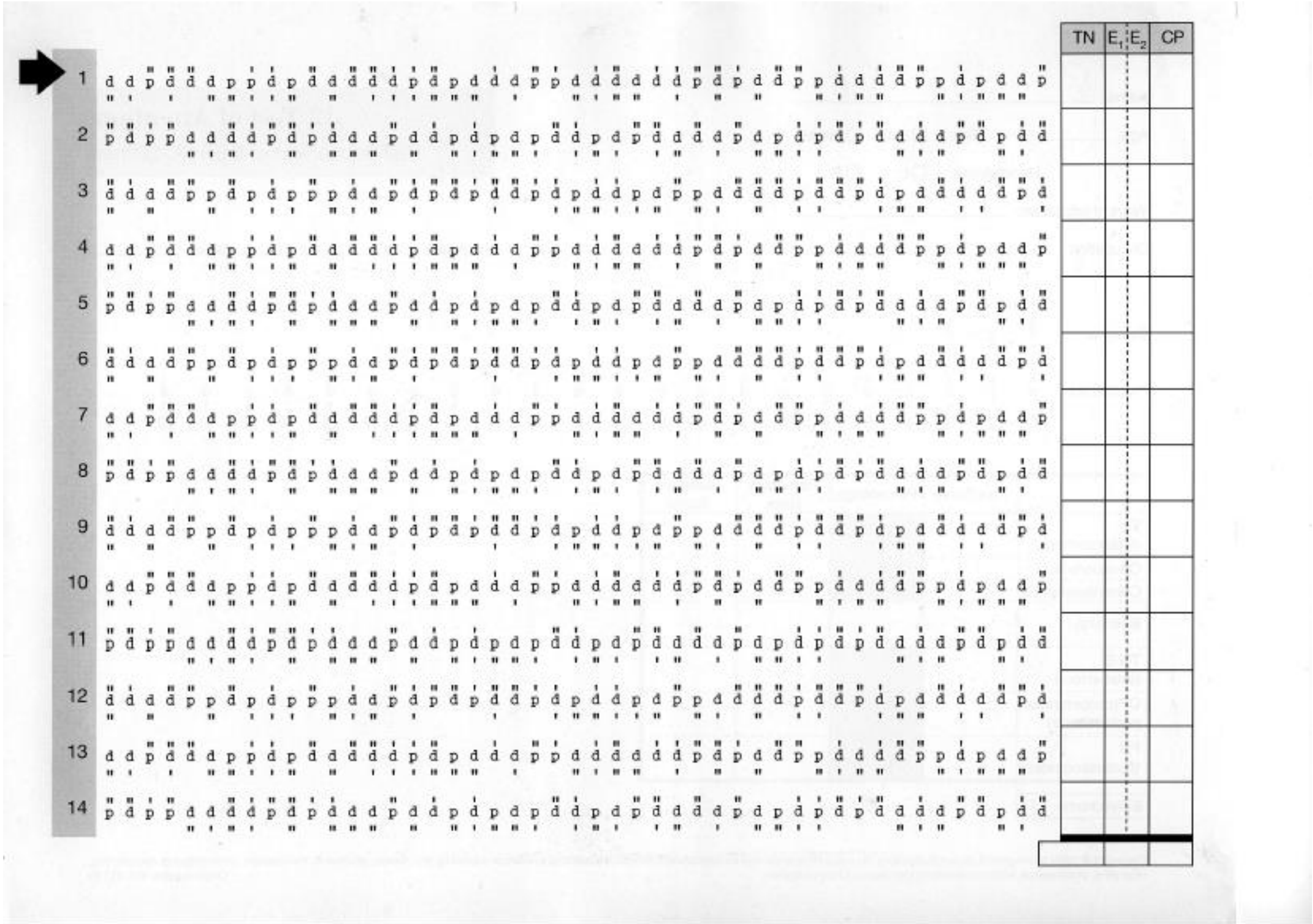

Time of completed _____ h ______ min

VISIT 4

Subject Initials ☐☐☐ **Subject ID** ☐☐☐☐☐ **Date:** ☐☐ / ☐☐ / ☐☐
Month Day Year

Subject Off Study

Date subject went Off Study: ☐☐ / ☐☐ / ☐☐
Month Day Year

Last visit completed

INDICATE OFF STUDY REASON: (select only one)

☐[1] Study Activities Completed

If the subject was withdrawn prior to completing the study (i.e. early withdrawal), select one of the following:

☐[2] Subject withdrawn – by Subject PRIOR to enrollment**
☐[3] Subject withdrawn – by Subject AFTER enrollment**
☐[4] Subject withdrawn – by PI PRIOR to enrollment**
☐[5] Subject withdrawn – by PI AFTER enrollment**
☐[6] Death
☐[7] Other**

If the subject was withdrawn, indicate specific reason(s): *(select all that apply and enter as text into the Subject Console > Follow-up > Subject Off Study Update > Explain field)*

☐[8] Subject lost to follow-up
☐[9] Subject refused follow-up
☐[10] Due to adverse events or complications
☐[11] Other**

**Additional explanation required:
(enter into the Subject Console > Follow-up > Subject Off Study Update > Explain field)

____
____
____

FORM COMPLETED BY: ____

Summary of patient's study outcome measures records

Table 6. Summary of patient's study outcome measures records

	Outcome assessment by*	Visit 1 Baseline	Visit 2	Visit 3	Visit 4
Nick Name	ObsRO				
Treatment code	ObsRO				
Gender (F/M)	ObsRO				
Age	ObsRO				
Acute phase duration	ClinRO				
Duration of decreased attention (d2-test)	PerfO				
Wisconsin URS Survey URTI Score	ClinRO				
Wisconsin URS Survey QOL Score	ClinRO				
Wisconsin URS Survey Total Score	ClinRO				
Duration and severity of fatigue	PRO				
Duration and severity of headache	PRO				
Duration of loss of smell	PRO				
Duration of loss of taste	PRO				
Duration and severity of rhinorrhea	PRO				
Duration of nasal congestions	PRO				
Duration and severity of sore throat	PRO				
Duration of Fever	PRO				
Duration and severity of cough	PRO				
Duration and severity pain in muscles, chest, and joints	PRO				
Duration of stay at home-hospital/sick listed	PRO				
Physical activity	PRO				
COVID-19 PCR test	BM				
Blood serum cytokin IL-6 (pg/ml)	BM				
D-Dimer	BM				
C-reactive protein	BM				
Creatinine	BM				

* - COA, clinical outcome assessment; ClinRO, clinician-reported outcome; ObsRO, observer-reported outcome; PerfO, performance outcome; PRO, patient-reported outcome, BM – biomarker.

Form Completed By: ________________________________**Date:** ______________

Site PI Signature: ________________________________Date: _____________

Subject Initials				Subject ID#						Page			of		

Adverse Event Tracking Log

Insert question to ask participant (e.g. Were there any adverse events? Y/N)

#	Date Reported	Adverse Event Description	Adverse Event Category**	Start Date	End Date	Ongoing (Y or N)	Outcome[1]	Severity/ Grade[2]	Serious (Y or N)	Expected (Y or N)	AE Treatment[3]	Action Taken[4]	Attribution[5]	PI Initials	Date of PI Initials

# - AE number. "1" indicates the first adverse event documented on the form, 2 = the second, etc. If the adverse event changes in severity, enter it as a separate adverse event row on the paper form using the same AE number as the one that ended.

Subject Initials				Subject ID						Page			of		

Outcome[1]
0 – Fatal
1 – Not recovered/not resolved
2 – Recovered w/sequelae
3 – Recovered w/o sequelae
4 – Recovering/Resolving

Severity/Grade[2]
1 – Mild
2 – Moderate
3 – Severe

AE Treatment[3]
0 – None
1 – Medication(s)
2 – Non-medication TX

Action Taken[4] with Study Intervention
0 – None
1 – Interrupted
2 – Discontinued
3 – Dose reduced
4 – Dose increased

Attribution/ Relatedness[5]
0 – Definite
1 – Probable
2 – Possible
3 – Unlikely
4 – Unrelated

Drug Dispensation & Accountability Log

		Dispensed to Subject					Returned by Subject				Compliance		
Lot Number(s)	Randomization Number	Number of Containers Dispensed	Number of Units Dispensed	Date Dispensed	Dispensed by (staff initials)	Expected Date of First Dose	Number of Containers Returned	Number of units returned	Date Returned	Verified by (staff initials)	*Actual* number of units used/taken	*Estimated* number of units to be used/taken	% compliance*

*Calculation of % Compliance = Actual taken / Estimated to be taken

Form Completed By: ____ **Date:** _________________

Site PI Signature: ____ Date: ________________

PROTOCOL DEVIATION FORM	
**Subject Random No: __ __ __ __**	**Subject Initials : __ __ __**

DEVIATION CODES			
A – Consent Procedures<br>B – Inclusion/Exclusion Criteria<br>C – Concomitant Medication/Therapy<br>D – Laboratory Assessments/Procedures	E – Study Procedures<br>F – Serious Adverse Event Reporting / Unanticipated Adverse Device Effect	G – Randomization Procedures/Study Drug Dosing<br>H – Visit Schedule/Interval	I – Efficacy Ratings<br>J – Other

SL No:	Date of Deviation occured	Date of Deviation identified	Deviation Description	Dev. Type	Affects subject participation	EC reporting required
	__ __ / __ __ __ / __ __	__ __ / __ __ __ / __ __			Yes No	Yes No

Data verified by :	Signature :

PROTOCOL VIOLATION FORM	
**Subject Random No: __ __ __ __**	**Subject Initials : __ __ __**

VIOLATION CODES		
A. Screening before taking ICF<br>B. Improper Informed consent<br>C. Missing ICF<br>D. Improper Inclusion of subjects	E. Breach of confidentiality<br>F. Falsification of records<br>G. Wrong treatment allocation<br>H. Subject withdrawal criteria not followed	I. Omission of evaluations / tests<br>Improper use of concomitant medications

**SL No:**	**Date of Violation occurred**	**Date of Violation identified**	**Violation Description**	**Violation Type**	**Affects subject participation**	**EC reporting Done**
	__ __ / __ __ __ / __ __	__ __ / __ __ __ / __ __			Yes No	Yes No

Data verified by :	Signature:

SAE			
**Subject Screening No: __ __ __ __ __**			**Subject Initials : __ __ __**
SAE report:#<br><br>Type of report: Initial Follow up<br><br>Final	Date of Submission to Sponsor:<br><br>Date of Submission to EC:		
**Reason for Reporting**	**Yes**	**No**	
Death			Date:
Life-threatening event?			
In-patient hospitalisation or prolongation of existing hospitalisation?			
Persistent or significant disability/ incapacity?			
Other pertinent medical reason for reporting?			
If other, please specify:			
Place of Occurrence:	Date of Onset :<br><br>Time (if known) :		
Brief Diagnosis:			
Causality Assessment: ☐ Unrelated to treatment ☐ Unlikely related ☐ Possibly related ☐ Probably related ☐ Definitely related			
Action taken with IP : ☐ None ☐ Dose reduced ☐ Discontinued			
Treatment Action : ☐ Treated with medication ☐ Surgical Intervention ☐ other<br><br>If other describe :			
Withdrawn from the study ? ☐ Yes ☐ No	Expected SAE : ☐ Yes ☐ No		

Outcomes: ☐ Resolved ☐ Recovered with minor sequelae<br>treatment ☐ Recovered with major sequelae ☐ Ongoing / Continuing<br>☐ Condition worsening ☐ Death ☐ Unknown	Date of resolution<br>__ __ / __ __ __ / __ __
Name of the PI :<br>Site Name:<br>Address :<br>Contacts : Email :<br>Tel :<br>Signature : Date:	

Form Completed By: ______________________________________ **Date:** ______________

Site PI Signature: ______________________________________ Date: ______________
