# Supplementary material for: Efficacy of Kan Jang® in Patients with Mild COVID-19: Interim Analysis of a Randomized, Quadruple-Blind, Placebo-Controlled Trial
Source: Pharmaceuticals (Basel). 2022 Aug 17;15(8):1013. doi: 10.3390/ph15081013 (PMC9415141; doi:10.3390/ph15081013)
Supplement: Supplementary file 1 [file pharmaceuticals-15-01013-s001.zip › Supplement S5 CoA - Kan Jang andrographis batch 3400195.pdf]

Svenska Örtmedicinska Institutet
Swedish Herbal Institute

Certificate of analysis

Internal spec. no: SP.FV-

Product: Kan Jang Andrographis capsules

Batch no: 3400195 | Batch no. bulk: ……………………………

Extr. Andrographis: …………………………… | Pack. type: ……………………………

Extr. Eleutherococcus: …………………………… | Country: ……………………………

Parameter of analysis on packed	Method	Reference	Result Start	Result End
**Appearance**	Ocular	Transparent capsule with green/beige filling	Ej transparent vit kapsel	
**Loss on drying**	Ph. Eur. Curr. Ed. 2.8.17	≤ 7 %	4%	
**Diameter**	Caliper	7.6 mm ± 0.2 mm	7,5 mm	
**Length**	Caliper	21.7 mm ± 0.3 mm	21,4 mm	
**Uniformity of mass**	Ph. Eur. Curr. Ed. 2.9.5	Average = 555 mg ± 7.5%	525 mg	
**Uniformity of mass deviations**	Ph. Eur. Curr. Ed. 2.9.5	n ≤ 2 deviates 7.5-15%	2st	
		n = 0 deviates >15%		
**Disintegration**	Ph. Eur. Curr. Ed. 2.9.1	≤ 30 min	45 min	
**External**				
Microbial contamination	Ph. Eur. Curr. Ed. 5.1.8	Ph. Eur. Cat B		

Parameter of analysis on bulk	Method	Reference	Result
**Identity**	SM-010	= ref	= Ref
**Assay: UPLC** - Andrographolide	SM-125	-	13,8 mg/capsule
**UPLC** - 14-Deoxy-11,12-didehydroandr.	SM-125	-	1,8 mg/capsule
∑ Andrographolides	SM-125	12 - 18 mg/capsule	16 mg/capsule
**UPLC** - Neoandrographolide	SM-125	n.a.	3,9 mg/capsule
**Assay: HPLC** - Eleutheroside B	SM-066	-	0,12 mg/capsule
**HPLC** - Eleutheroside E	SM-066	-	0,13 mg/capsule
Σ Eleutheroside B and E	SM-066	0.15-0.51 mg/capsule	0,25 mg/capsule
**Amount of native extract**			
Eleutherococcus	SM-136	37.5 mg/capsule ±10%	
Androgrographis	SM-136	375 mg/capsule ±10%	
**External**			
Pyrrolizidine alkaloids	SOP 805521 Phytolab	≤ 0.25 µg/capsule*	

*With daily dose of 4 capsules

Date of analysis: …………………… | **Date of release:** ……………………
Analyst, sign: ……………/………… | **QP-responsible:** ……………………

**Comments:**	**Manufacturing date:**<br><br>**Expiry date (three years):**

Svenska Örtmedicinska Institutet

Swedish Herbal Institute

Kan Jang Capsule

HPTLC

3400195

Method: SM-010

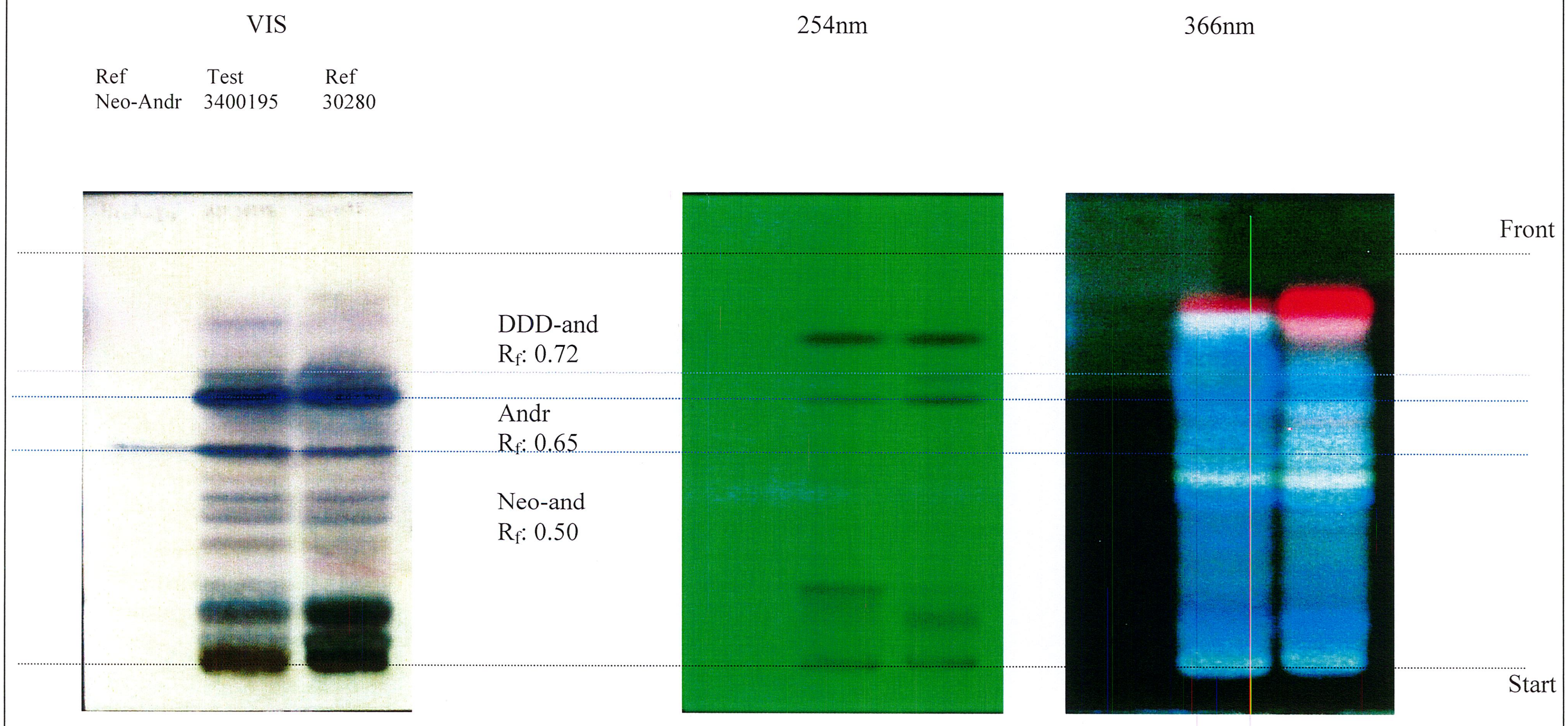

Absorbent:	Silica gel 60 F_{254} precoated HPTLC plates
Solvent system:	Etylacetate : methanol : water (38 : 7 : 4)
Spray reagents:	vanillin-sulphuric acid solution

Sign: [signature]

Date: 2021-05-06

Mikrolab Stockholm AB

SWEDAC ACKREDITERING
Ackred. nr 2028
Provning
ISO/IEC 17025

Rapport
Utfärdat av ackrediterat laboratorium

Svenska Örtmedicinska Ins AB

Susanna Panosyan
Kövlingevägen 21
312 50 Vallberga

Datum	Kundnummer	Provnummer
2021-04-28	20031	**K2102825-00**

PROVUPPGIFTER

Produkt:	Kanjang
Artikelnummer:	N/A
Batchnummer:	3400195
Undersökningstyp:	Totalantal odling/fasta produkter
Ankomstdatum:	2021-04-23
Testdatum:	2021-04-23
Testad mängd:	45g
Analyskrav enligt:	Se metod
Provtagare:	Håkan Lejon

Capsule.

**ANALYSRESULTAT**	Metod	Resultat	Enhet
Aeroba bakterier	M0002	<10	CFU/g
Mögelsvampar	M0002	<10	CFU/g
Jästsvampar	M0002	<10	CFU/g
Escherichia coli	M0017	Ej påvisad	/1g
G-neg. bakterier	M0016	<10	CFU/g
Salmonella species	M0018	Ej påvisad	/25 g

UTLÅTANDE OCH UPPLYSNINGAR

TAMC: <10 CFU/g
TYMC: <10 CFU/g
Analys enligt gällande EP 2.6.12/2.6.13 och USP <61>/<62>

Sollentuna
Datum: 2021-04-28

Susanna Nilsson
Analysansvarig

Linda Storbjörk
Laboratorieansvarig

** Efter metodbeteckning, anger att metoden ej är ackrediterad.*

Resultaten avser endast testade produkter.

Upplysningar om mätosäkerhet för kvantitativa mikrobiologiska analyser lämnas på begäran.

Adress:
Mikrolab Stockholm AB
Kung Hans Väg 3
192 68 Sollentuna

Telefon
08-297900
E-post: sollentuna.lab@alsglobal.com

Styrelsens säte:
Sollentuna

Organisationsnummer
556794-2171

Empower 3 SOFTWARE

SampleName Kan Jang Andro Rapport tablett

Current Date 2021-05-06
Processing Method
Start Date 2021-05-05 16:13:06
System Name H_CLASS
Run Time 35.00 Minutes

Eleutherosid B
Eleutherosid E

Batch_No 3400195

Software Empower 3 Software Build 3471
Channel PDA_210.0nm, PDA_220.0nm
Tablet_Weight
Column no.: 427571

Name: Eleutherosid B

	SampleName	Batch_No	Injection Volume (ul)	SampleWeight	RT	Area	Height	Name	Procent_Amount
1	Kan Jang Andro	3400195	10.00	30.18800	5.368	397829	32101	Eleutherosid B	0.0223
2	Kan Jang Andro	3400195	10.00	30.18800	5.485	374764	27891	Eleutherosid B	0.0210
3	Kan Jang Andro	3400195	10.00	30.18800	5.499	412101	32830	Eleutherosid B	0.0231
Mean					5.450				0.0221
Std. Dev.					0.07				0.0010
% RSD					1.3				4.75

Name: Eleutherosid E

	SampleName	Batch_No	Injection Volume (ul)	SampleWeight	RT	Area	Height	Name	Procent_Amount
1	Kan Jang Andro	3400195	10.00	30.18800	16.980	572241	39768	Eleutherosid E	0.0242
2	Kan Jang Andro	3400195	10.00	30.18800	17.016	574688	40088	Eleutherosid E	0.0243
3	Kan Jang Andro	3400195	10.00	30.18800	17.053	570466	39164	Eleutherosid E	0.0241
Mean					17.016				0.0242
Std. Dev.					0.04				0.0001
% RSD					0.2				0.37

Results	*mg/tablet*	*calculated*	*% found*
Eleutherosid B:	0,12 mg/capsule		
Eleutherosid E:	0,13 mg/capsule		
Eleutherosides:	0,25 mg/capsule		

Sig.: [signature]

Chromatogram

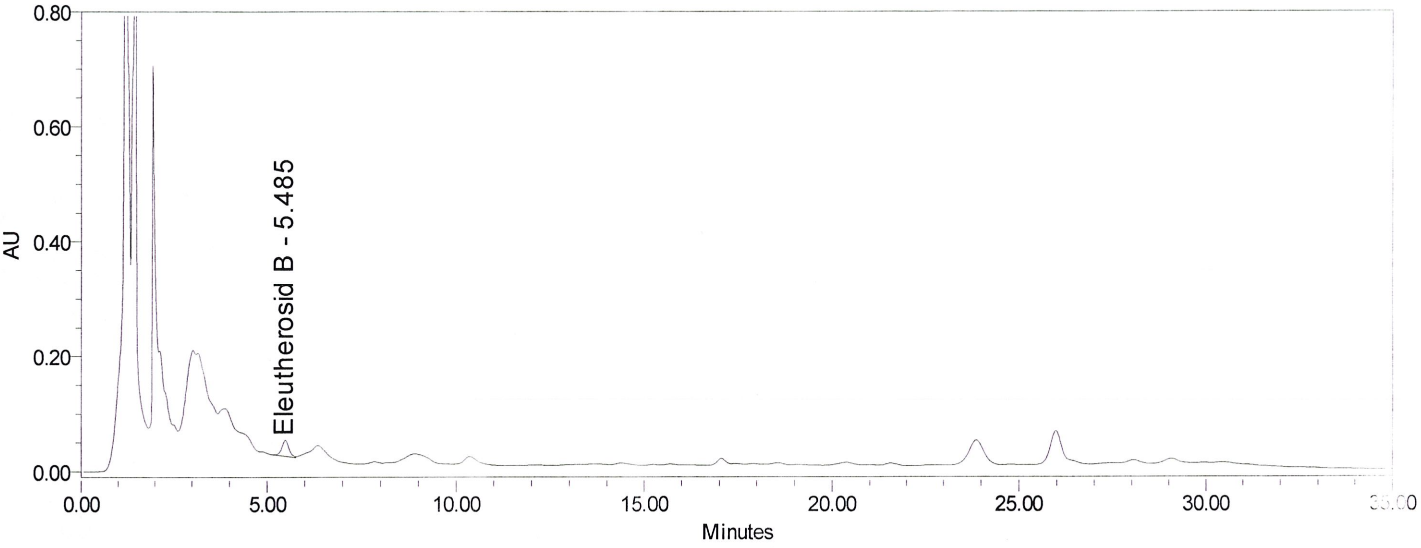

SampleName Kan Jang Andro; Batch_No 3400195; Vial 1:A,3; Date Acquired 2021-05-05 22:51:35 CEST; Channel Name PDA_220.0nm

Chromatogram

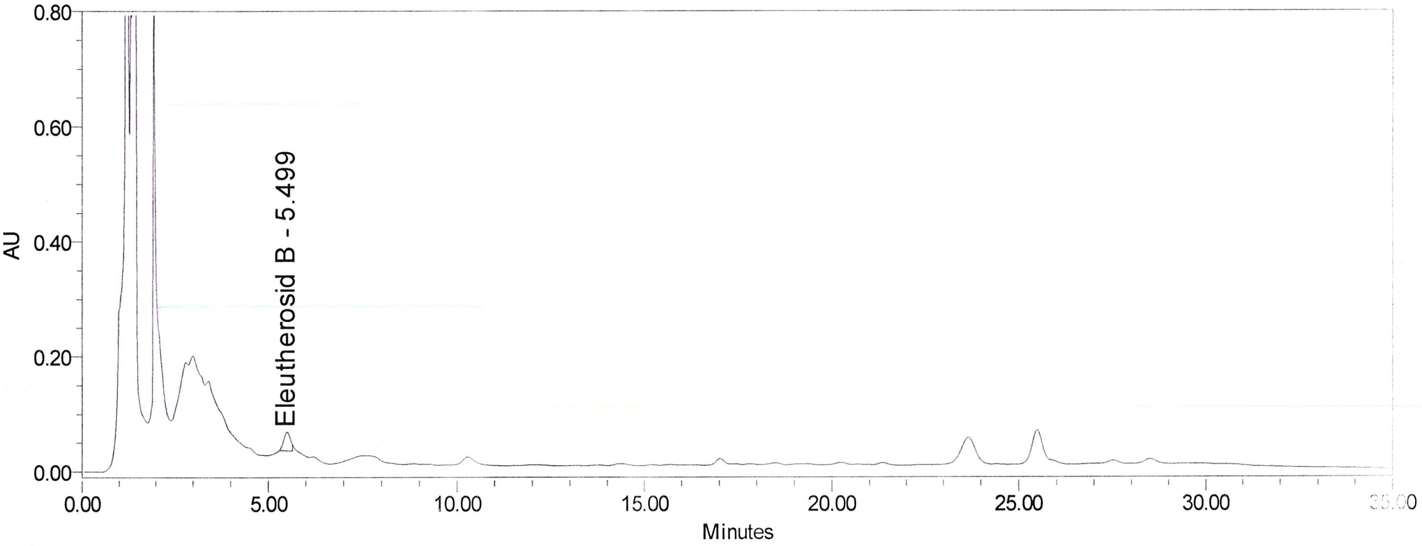

SampleName Kan Jang Andro; Batch_No 3400195; Vial 1:A,3; Date Acquired 2021-05-05 23:52:43 CEST; Channel Name PDA_220.0nm

Sig.: ______

Chromatogram

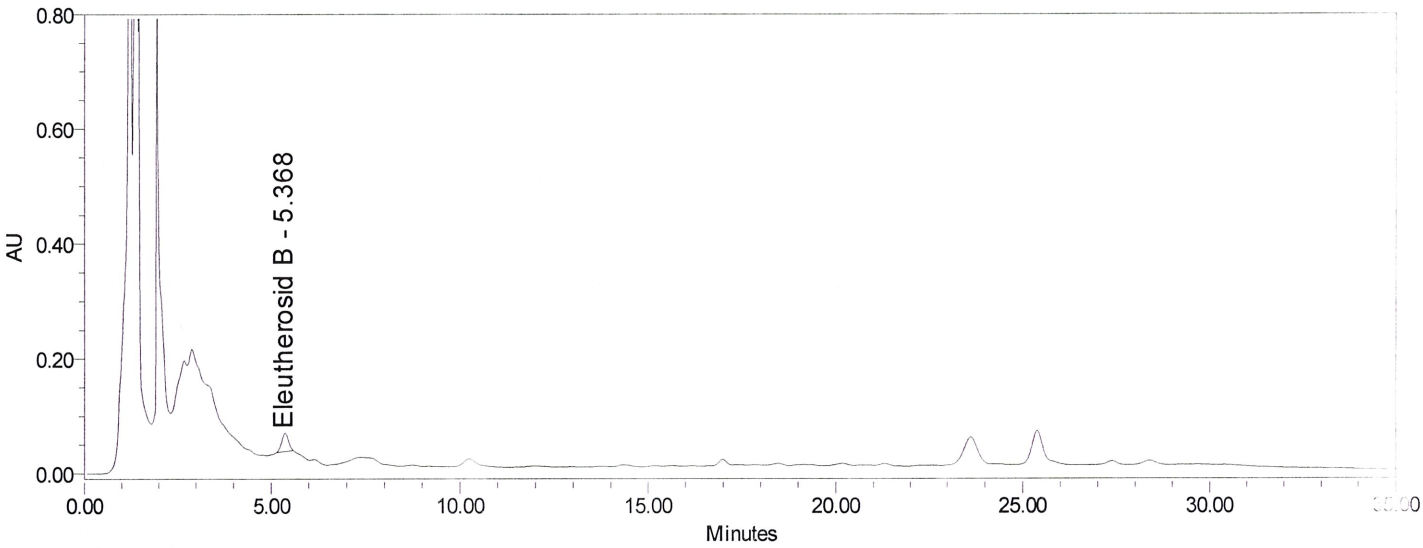

SampleName Kan Jang Andro; Batch_No 3400195; Vial 1:A,3; Date Acquired 2021-05-06 00:53:53 CEST; Channel Name PDA_220.0nm

Chromatogram

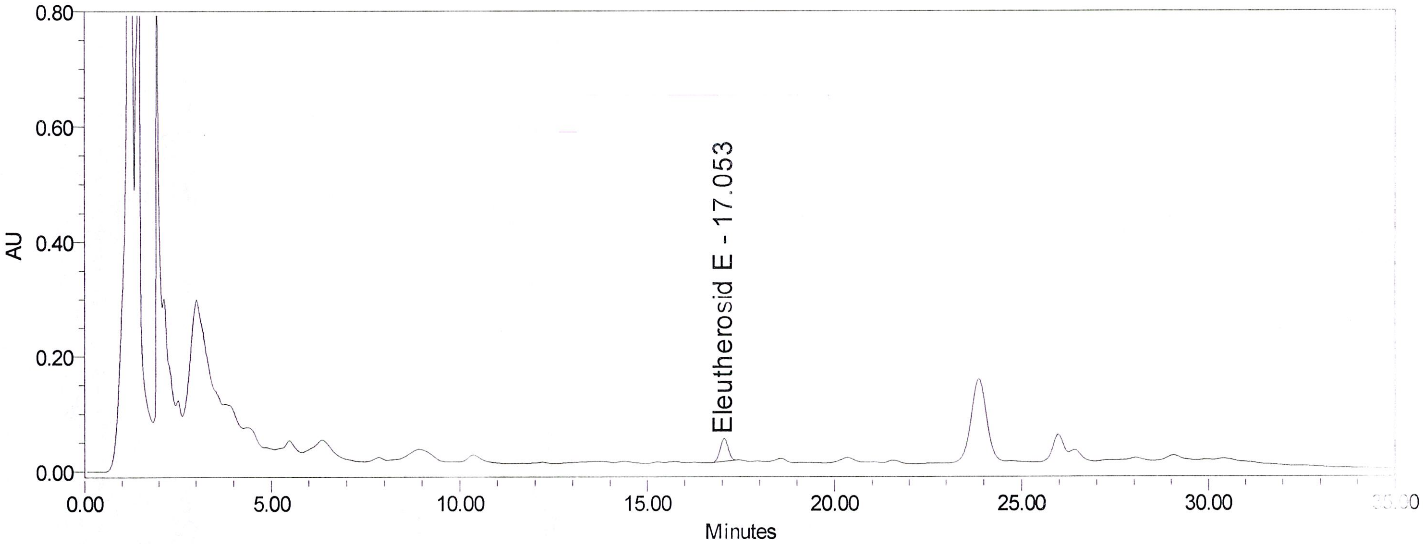

SampleName Kan Jang Andro; Batch_No 3400195; Vial 1:A,3; Date Acquired 2021-05-05 22:51:35 CEST; Channel Name PDA_210.0nm

Sig.:

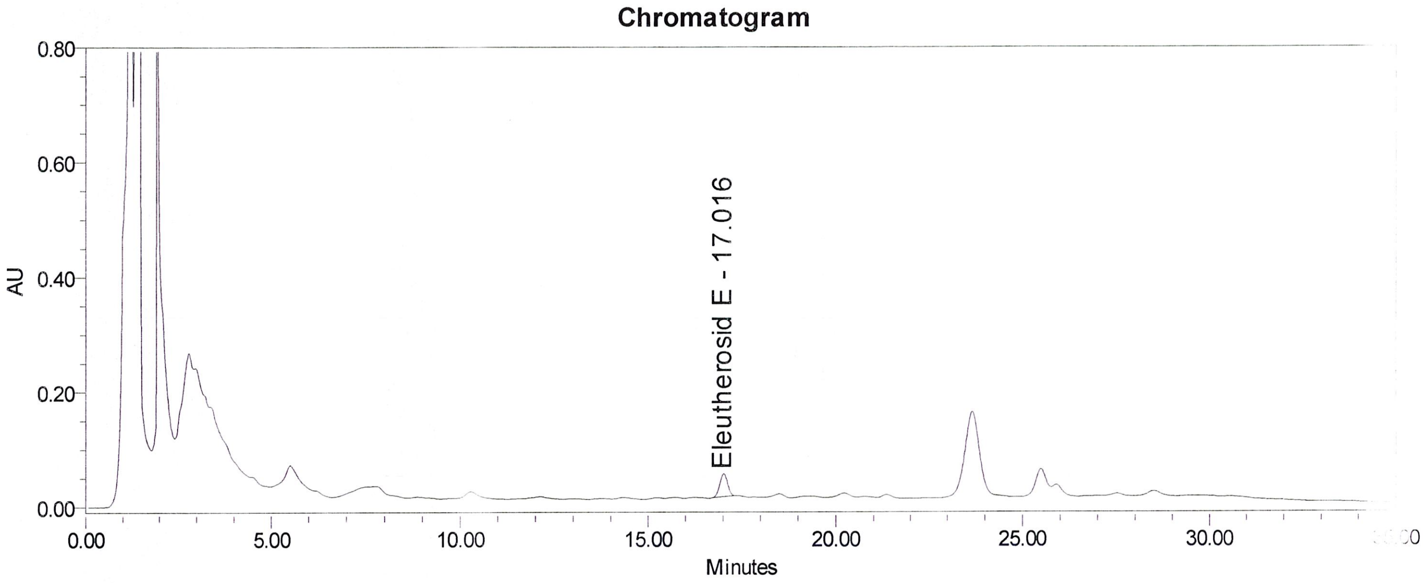

SampleName Kan Jang Andro; Batch_No 3400195; Vial 1:A,3; Date Acquired 2021-05-05 23:52:4[illegible] CEST; Channel Name PDA_210.0nm

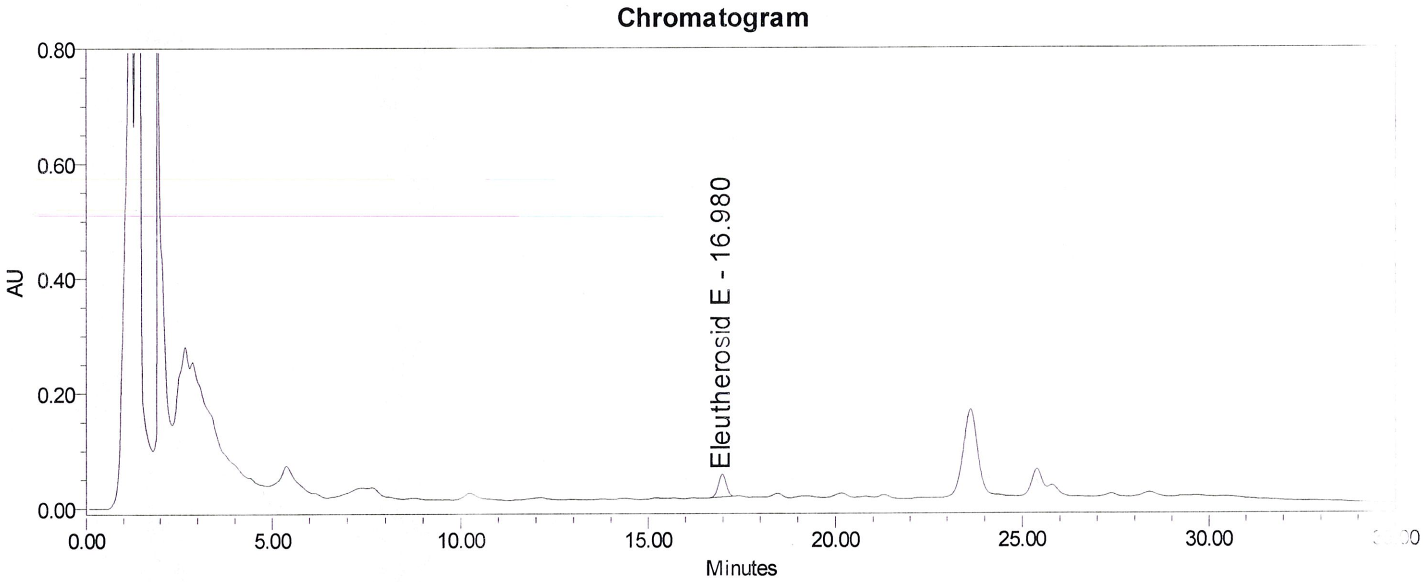

SampleName Kan Jang Andro; Batch_No 3400195; Vial 1:A,3; Date Acquired 2021-05-06 00:53:5[illegible] CEST; Channel Name PDA_210.0nm

Sig.: [illegible]

Second identification with PDA

rocessing Method Eleuth_PDA
un Time 35.00 Minutes
tart Date 2021-05-05 16:13:06

Eleutherosid B, Eleutherosid E

Current Date 2021-05-06
Software Empower 3 Software [illegible]
System Name H_CLASS

SampleName Kan Jang Andro

Batch_No 3400195

Spectrum Index Plot

Eleutherosid E - 17.053

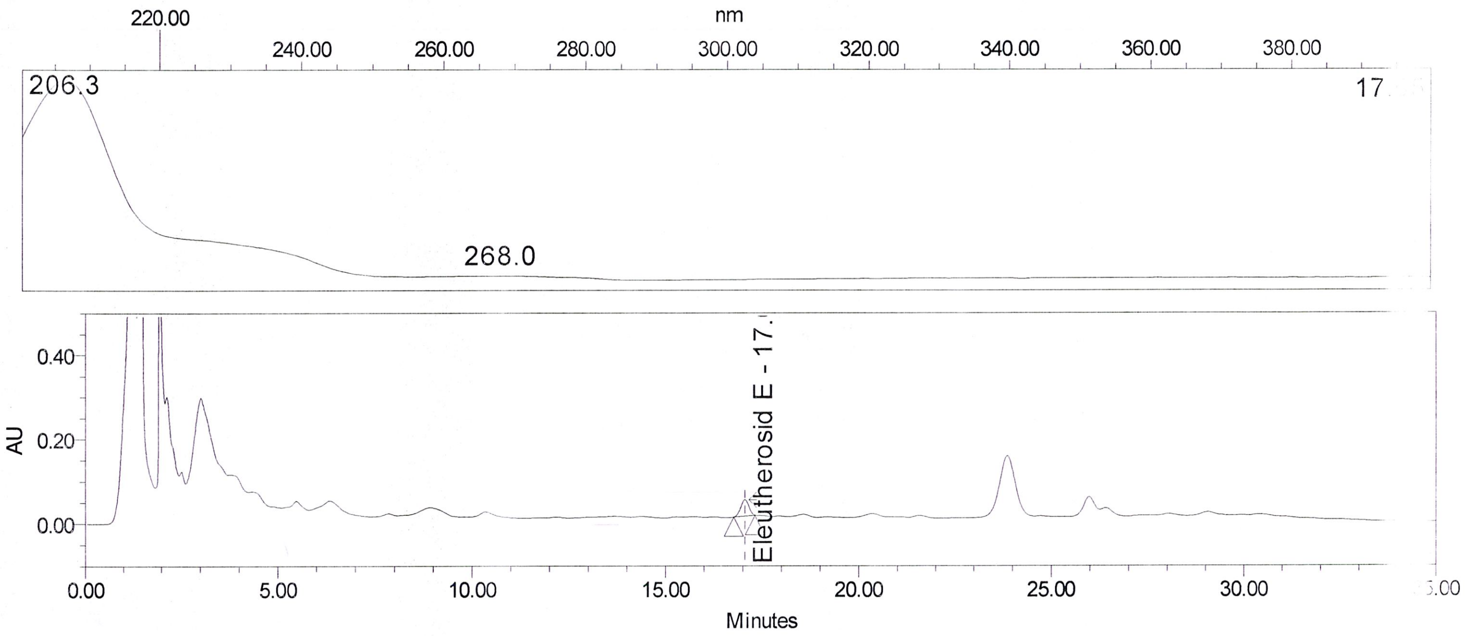

Spectrum Index Plot

Eleutherosid B - 5.483

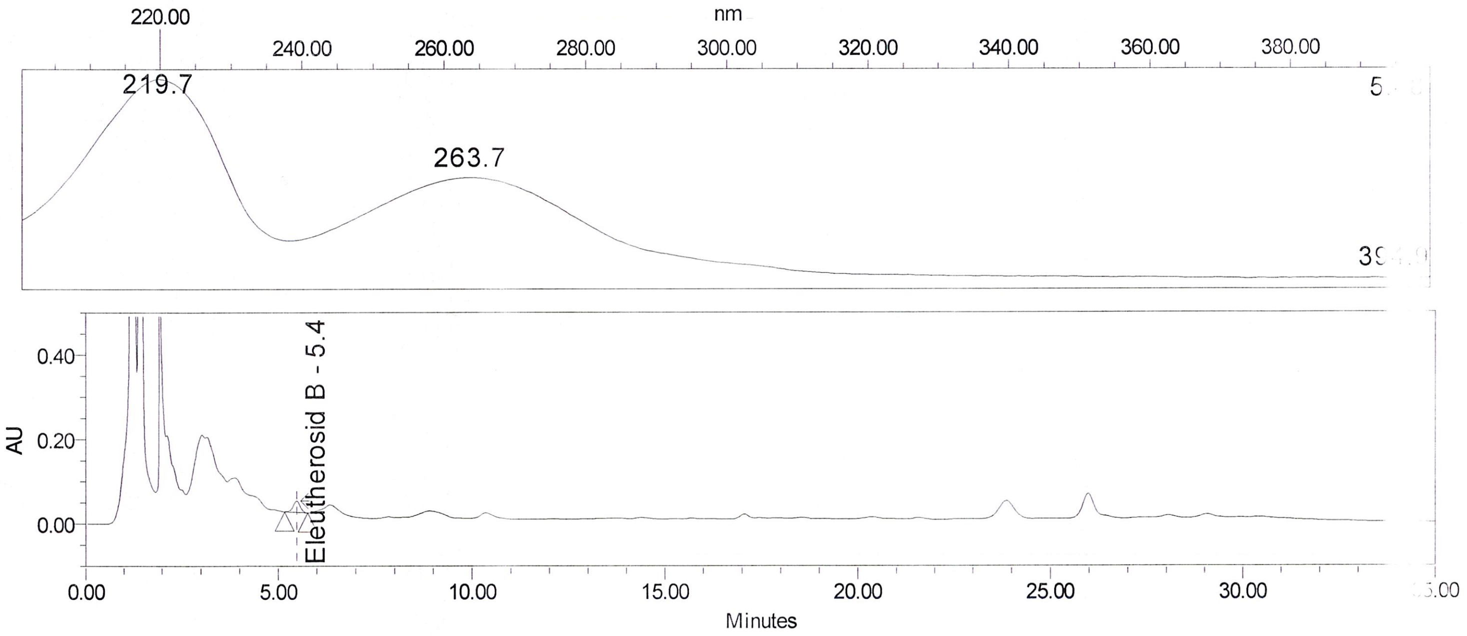

Sign.: [signature]

Match Plot

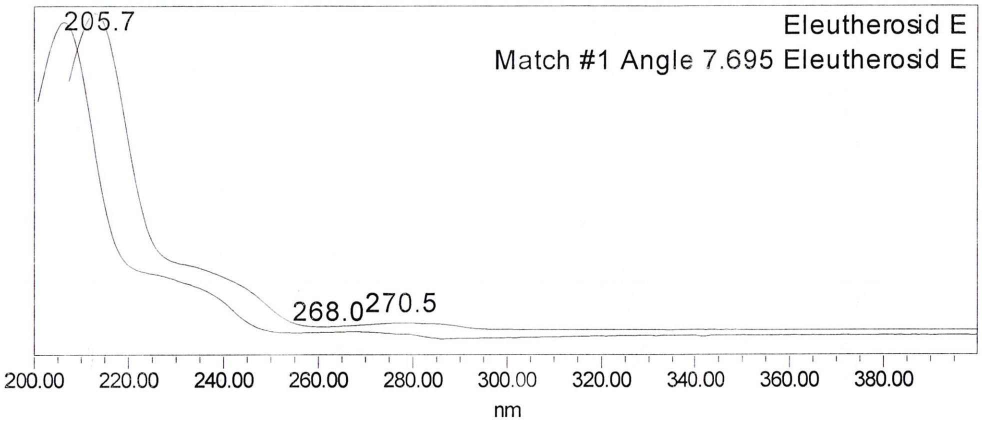

SampleName Kan Jang Andro Batch_No 3400195 Vial 1:A,3 Injection 1 Date Acquired 2021-05-05 22:51:35 CEST Name Eleutherosid E Match1 Angle 7.695 Match1 Threshold 90.000 Match1 Spect. Name Eleutherosid E

Match Plot

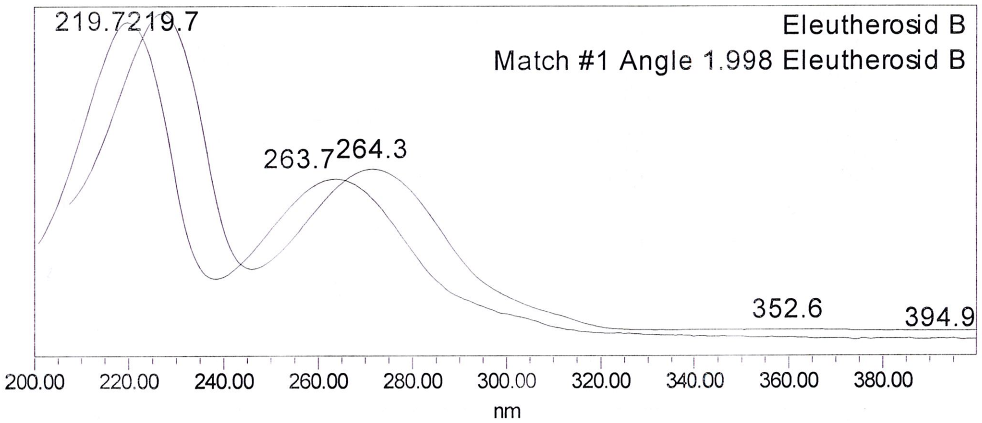

SampleName Kan Jang Andro Batch_No 3400195 Vial 1:A,3 Injection 1 Date Acquired 2021-05-05 22:51:35 CEST Name Eleutherosid B Match1 Angle 1.998 Match1 Threshold 52.885 Match1 Spect. Name Eleutherosid B

PDA Result Table

	Name	RT	Purity1 Angle	Purity1 Threshold	Match1 Spect. Name	Match1 Angle	Match1 Threshold
1	Eleutherosid B	5.483	5.239	90.000	Eleutherosid B	1.998	52.885
2	Eleutherosid E	17.053	6.078	90.000	Eleutherosid E	7.695	90.000

Sign.: [signature]

Purity Plot

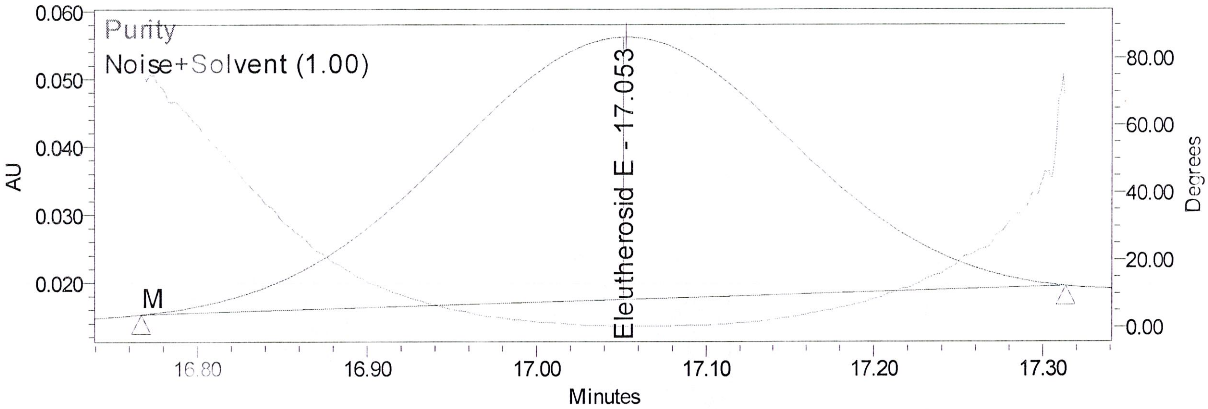

PA: 6.078 TH: 90.000

SampleName: Kan Jang Andro Batch_No 3400195 Vial: 1:A,3 Injection: 1 Date Acquired: 2021-05-05 22:51:35 CEST Purity1 Angle: 6.078 Purity1 Threshold: 90.000

Purity

Noise+Solvent (1.00)

Purity Plot

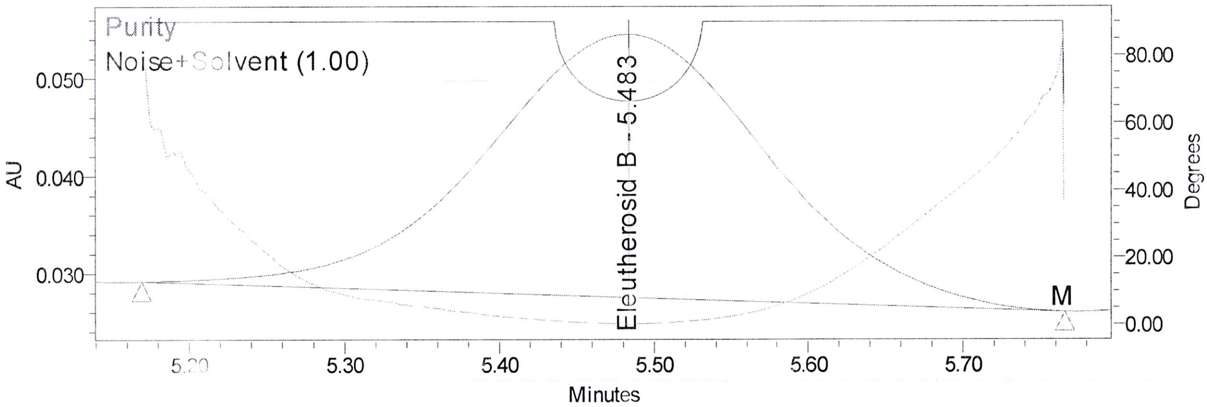

PA: 5.239 TH: 90.000

SampleName: Kan Jang Andro Batch_No 3400195 Vial: 1:A,3 Injection: 1 Date Acquired: 2021-05-05 22:51:35 CEST Purity1 Angle: 5.239 Purity1 Threshold: 90.000

Purity

Noise+Solvent (1.00)

Sign.: [signature]

Empower 3 SOFTWARE

Processing Method Andrographis
Start Date 2021-05-05 09:47:18 CEST
Tablet_weight:

SampleName Kan Jang

Name Andrographolide, Neoandrographolide, Deoxy-didehydroandrographolide

Batch_No 3400195

Current Date 2021-05-05
Software Empower 3 Software Build 347
Column no.: Acquity UPLC BEH C18 1.7μ
01773000715504
Channel 205 nm, 254 nm, 223
Run Time 5.00 Minutes

Component Results
Name: Andrographolide

	SampleName	Batch_No	SampleWeight	RT	Area	Height	Name	Percent_amount
1	Kan Jang	3400195	3.88400	2.077	686188	293954	Andrographolide	2,625
2	Kan Jang	3400195	3.88400	2.079	686984	294074	Andrographolide	2,628
3	Kan Jang	3400195	3.88400	2.081	688935	294607	Andrographolide	2,635
Mean				2.1				2,629
Std. Dev.				0.0				
% RSD				0.1				

Component Results
Name: Neoandrographolide

	SampleName	Batch_No	SampleWeight	RT	Area	Height	Name	Percent_amount
1	Kan Jang	3400195	3.88400	3.033	162998	128980	Neoandrographolide	0,738
2	Kan Jang	3400195	3.88400	3.034	163340	129369	Neoandrographolide	0,740
3	Kan Jang	3400195	3.88400	3.034	163731	129927	Neoandrographolide	0,742
Mean				3.0				0,740
Std. Dev.				0.0				
% RSD				0.0				

Component Results
Name: Deoxy-didehydroandrographolide

	SampleName	Batch_No	SampleWeight	RT	Area	Height	Name	Percent_amount
1	Kan Jang	3400195	3.88400	3.315	71523	39793	Deoxy-didehydroandrographolide	0,342
2	Kan Jang	3400195	3.88400	3.317	71383	39808	Deoxy-didehydroandrographolide	0,341
3	Kan Jang	3400195	3.88400	3.317	71387	39914	Deoxy-didehydroandrographolide	0,341
Mean				3.3				0,342
Std. Dev.				0.0				
% RSD				0.0				

Results	mg/tbl	calculated	%found
**Andrographolide:**	13,8 mg/tbl		
**Deoxy-11,12-didehydroandrographolide:**	1,8 mg/tbl		
**Total andr and DDD-andr:**	15,6 mg/tbl		
**Neoandrographolide:**	3,9 mg/tbl		

Sign.: [illegible]

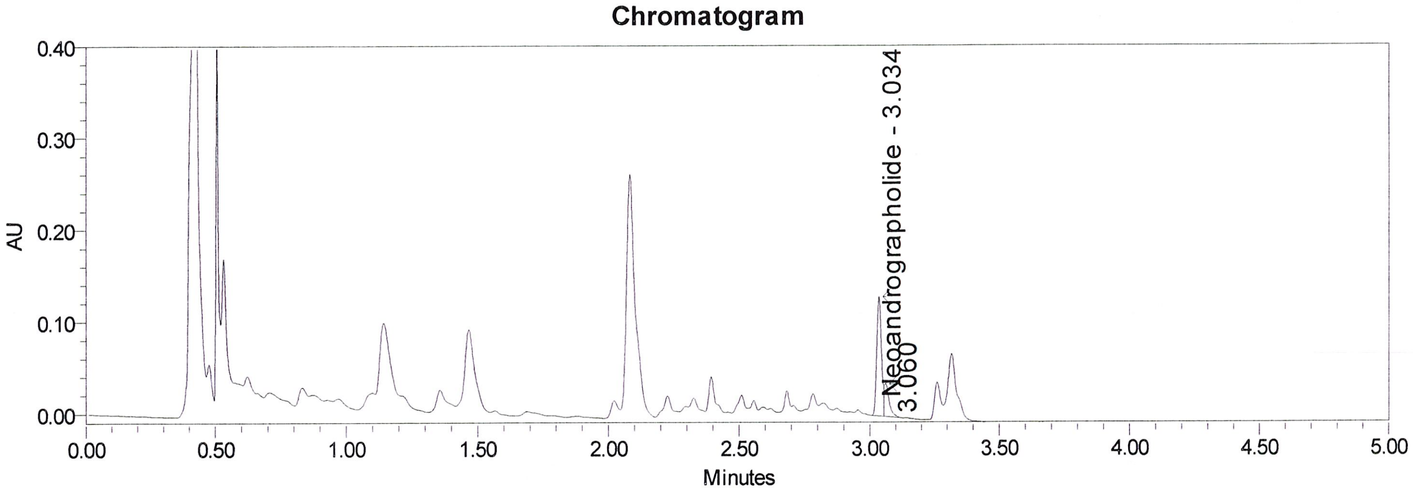

SampleName Kan Jang; Batch_No 3400195; Vial 1:A,1; Injection 1; Date Acquired 2021-05-05 09:49:02 CEST; Channel Name 205 nm

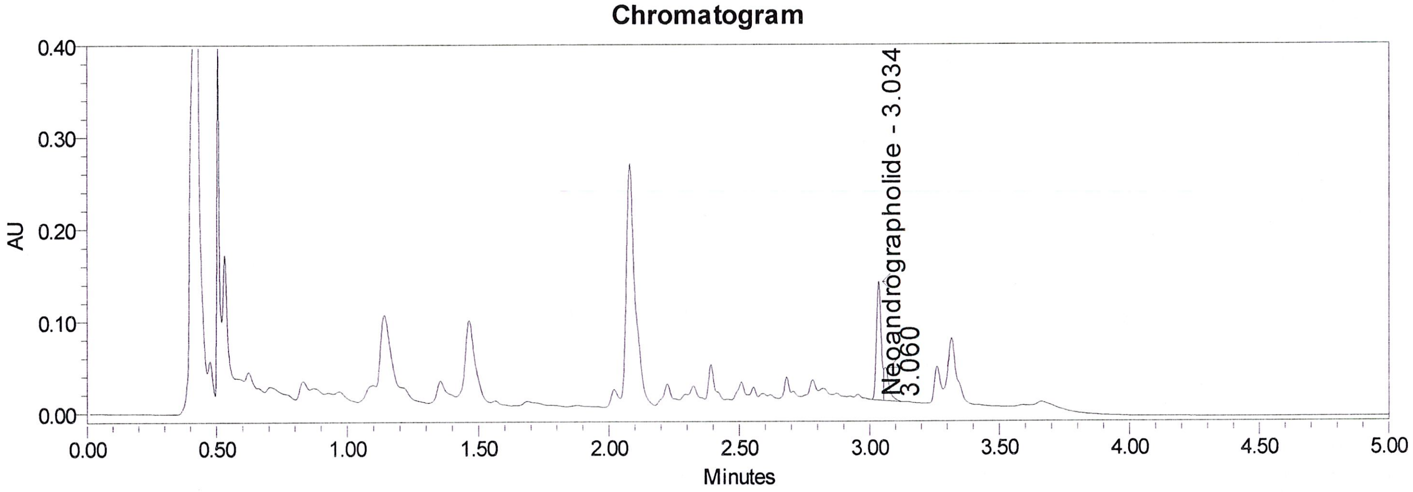

SampleName Kan Jang; Batch_No 3400195; Vial 1:A,1; Injection 2; Date Acquired 2021-05-05 09:54:53 CEST; Channel Name 205 nm

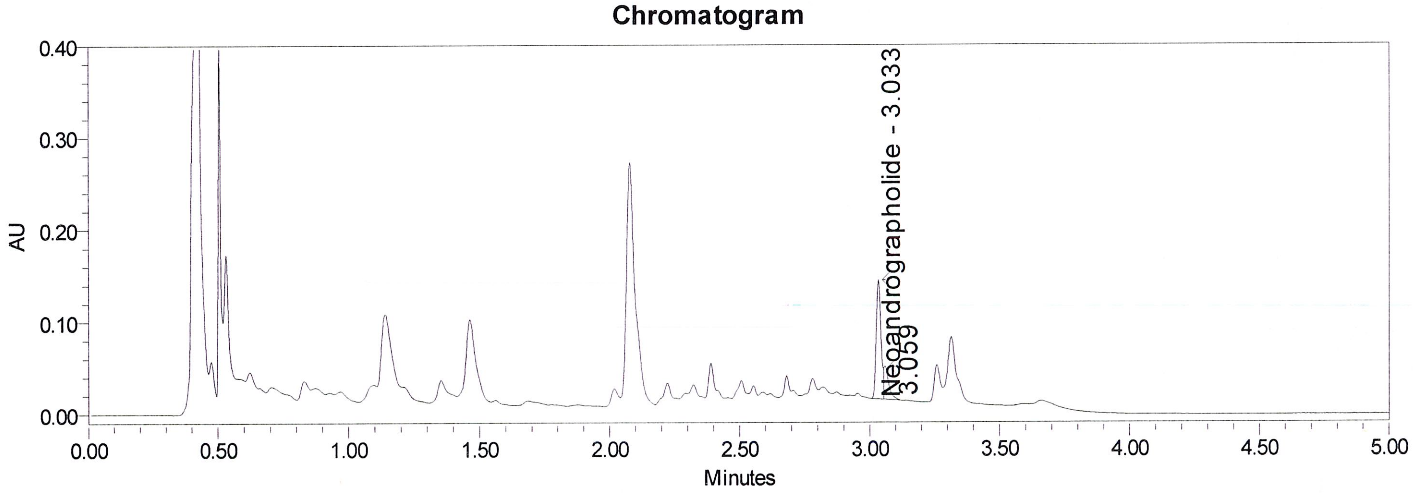

SampleName Kan Jang; Batch_No 3400195; Vial 1:A,1; Injection 3; Date Acquired 2021-05-05 10:00:44 CEST; Channel Name 205 nm

Sign.: ________

Chromatogram

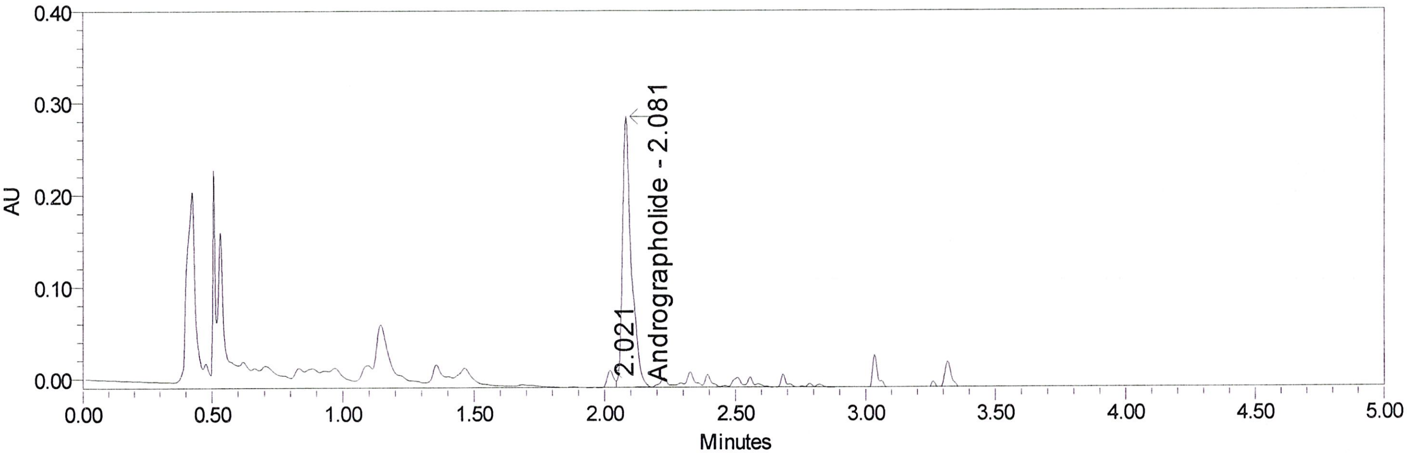

SampleName Kan Jang; Batch_No 3400195; Vial 1:A,1; Injection 1; Date Acquired 2021-05-05 09:49:02 CEST; Channel Name 223 nm

Chromatogram

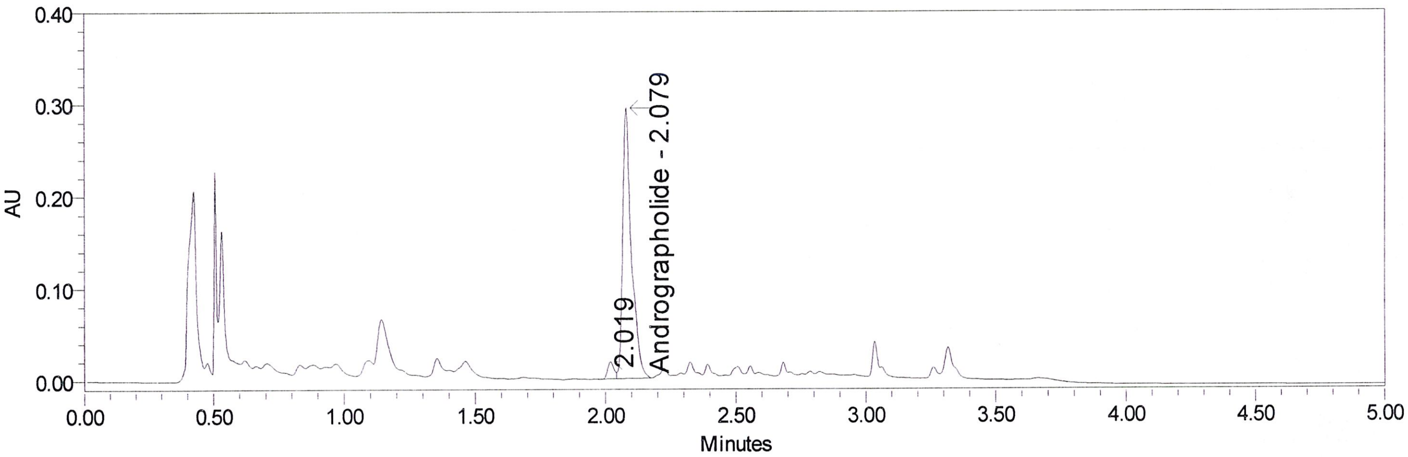

SampleName Kan Jang; Batch_No 3400195; Vial 1:A,1; Injection 2; Date Acquired 2021-05-05 09:54:53 CEST; Channel Name 223 nm

Chromatogram

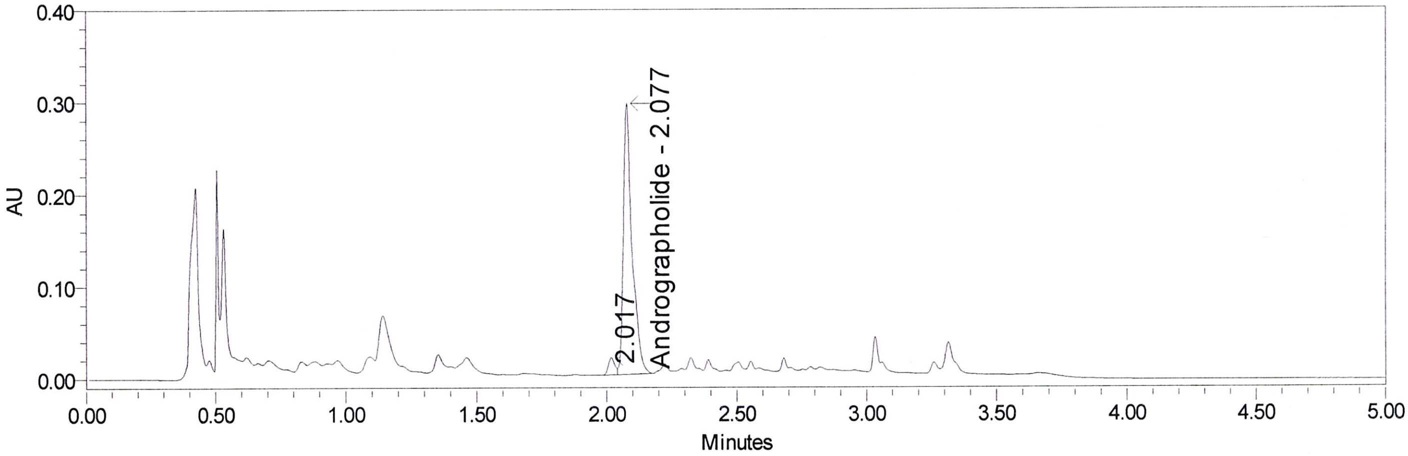

SampleName Kan Jang; Batch_No 3400195; Vial 1:A,1; Injection 3; Date Acquired 2021-05-05 10:00:44 CEST; Channel Name 223 nm

Sign.:

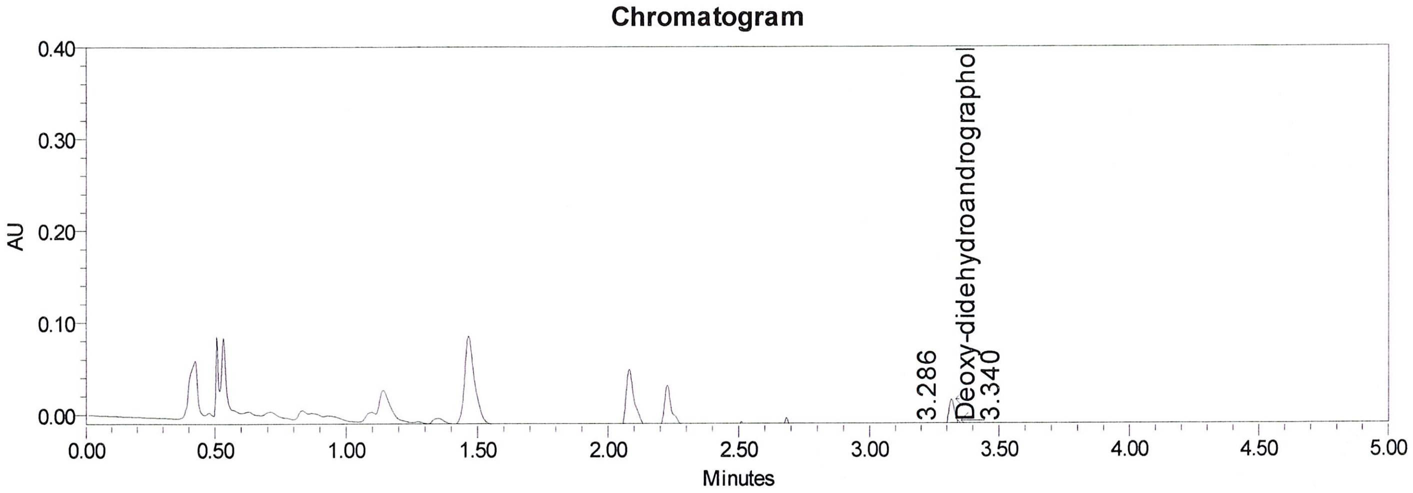

SampleName Kan Jang; Batch_No 3400195; Vial 1:A,1; Injection 1; Date Acquired 2021-05-05 09:49:02 CEST; Channel Name 254 nm

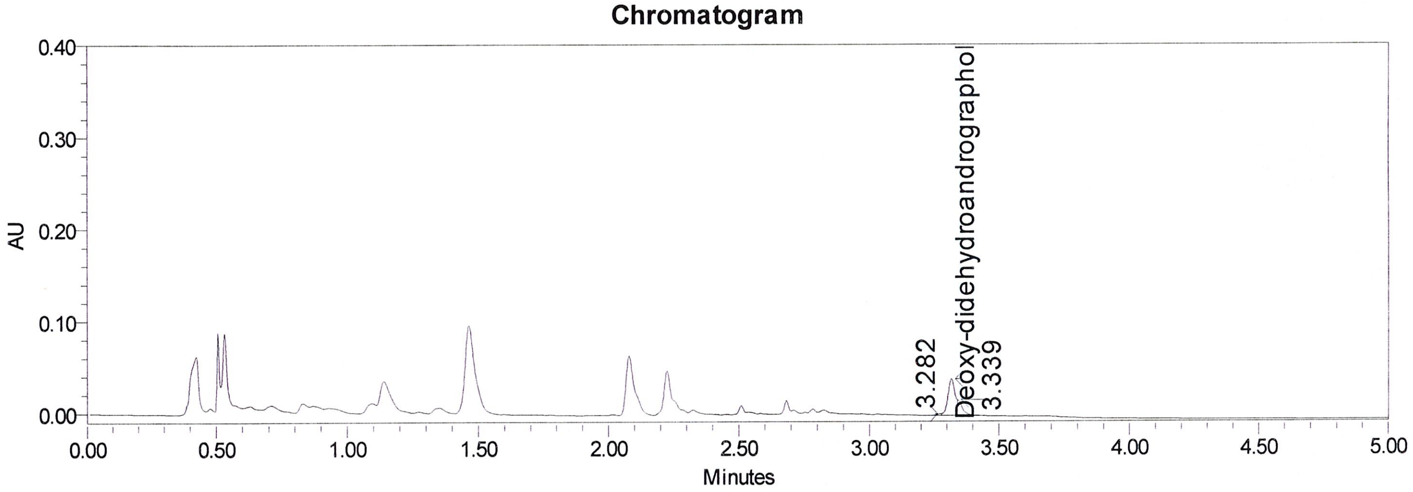

SampleName Kan Jang; Batch_No 3400195; Vial 1:A,1; Injection 2; Date Acquired 2021-05-05 09:54:53 CEST; Channel Name 254 nm

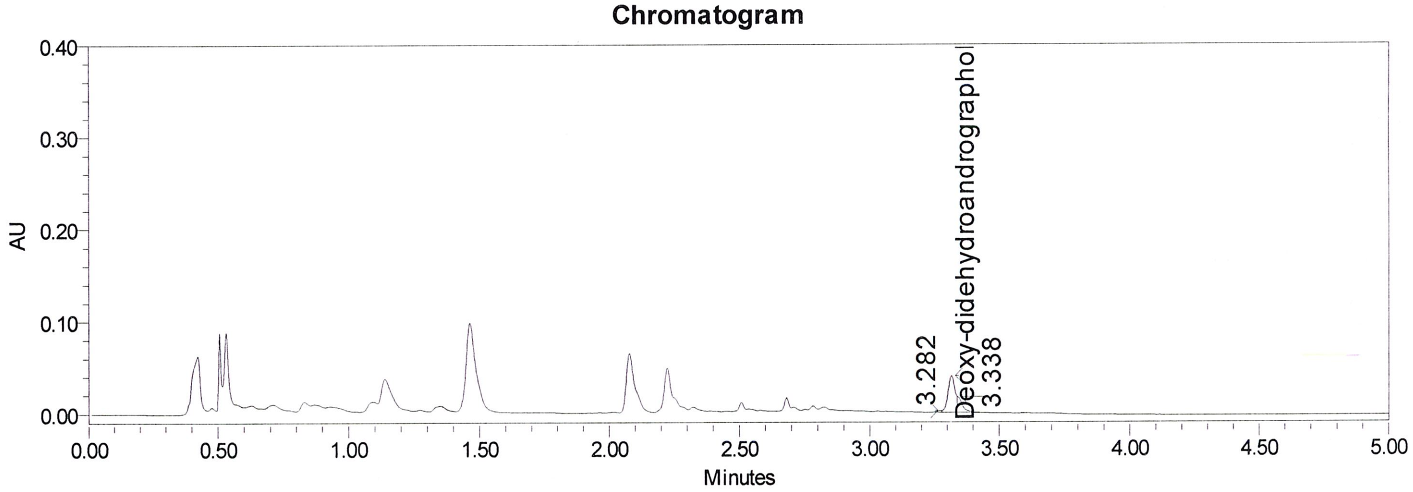

SampleName Kan Jang; Batch_No 3400195; Vial 1:A,1; Injection 3; Date Acquired 2021-05-05 10:00:44 CEST; Channel Name 254 nm

Sign.:

Sample Name: Kan Jang Andrographis
Batch No: 3400195

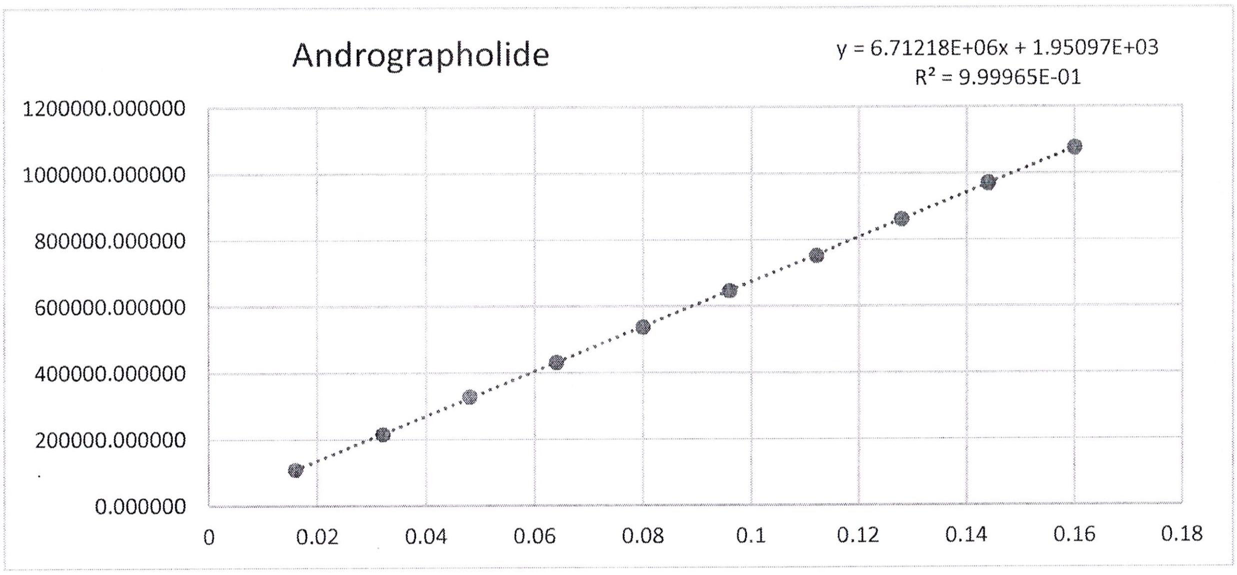

Injection		Sample Weight	Peakarea	Conc
	1	3.884	686188	2.625E-02
	2	3.884	686984	2.628E-02
	3	3.884	688935	2.635E-02
Average		3.884	687369	2.629E-02

Percent amount
2.629

Tablet weight: 525 mg

Tablet content: 13.80 mg/tab

Date: 2021-05-05

Sign: [signature]

Sample Name: Kan Jang Andrographis
Batch No: 3400195

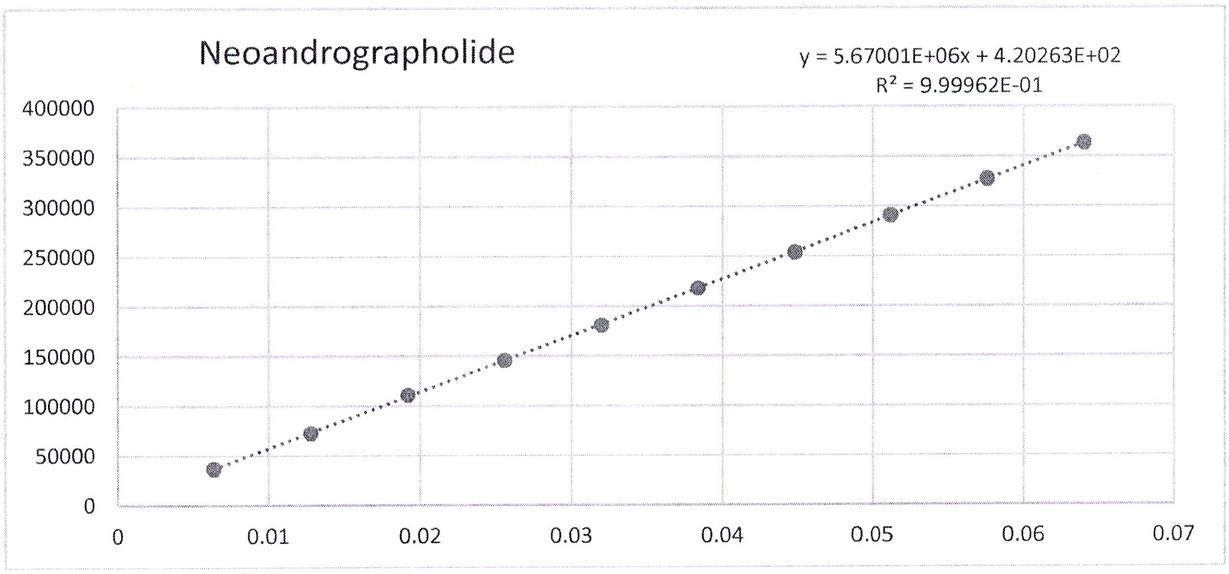

Injection		Sample Weight	Peakarea	Conc
	1	3.884	162998	7.382E-03
	2	3.884	163340	7.398E-03
	3	3.884	163731	7.416E-03
Average		3.884	163356	7.399E-03

Percent amount
0.740

Tablet weight: 525 mg

Tablet content: 3.88 mg/tab

Date: 2021-05-05

Sign: [signature]

Sample Name: Kan Jang Andrographis
Batch No: 3400195

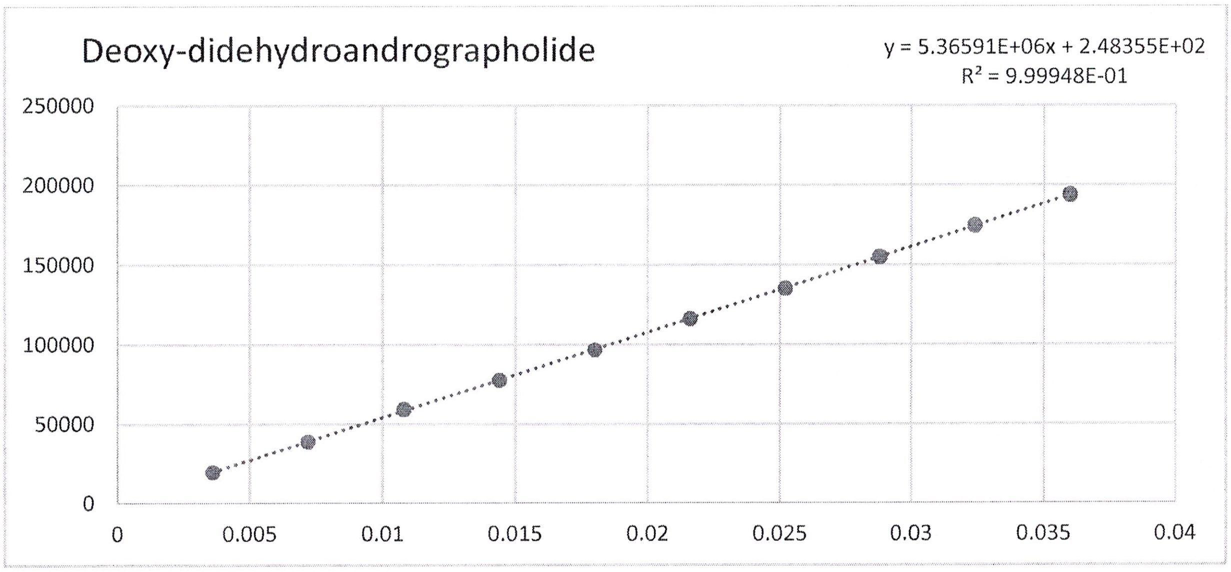

Injection		Sample Weight	Peakarea	Conc
	1	3.884	71523	3.42E-03
	2	3.884	71383	3.41E-03
	3	3.884	71387	3.41E-03
Average		3.884	71431	3.42E-03

Percent amount
0.342

Tablet weight: 525 mg

Tablet content: 1.79 mg/tab

Date: 2021-05-05

Sign: [signature]
